# High glucose promotes macrophage switching to the M1 phenotype via the downregulation of STAT-3 mediated autophagy

**DOI:** 10.1371/journal.pone.0314974

**Published:** 2024-12-31

**Authors:** Yu Zhao, Yuteng Jiang, Fengmei Wang, Li Sun, Mengyuan Ding, Liyuan Zhang, Beibei Wu, Xiaoliang Zhang

**Affiliations:** 1 Institute of Nephrology, Zhong Da Hospital, School of Medicine, Southeast University, Nanjing Jiangsu, China; 2 Department of Nephrology, Xuyi People’s Hospital, Huaian, China; 3 Department of Nephrology, The First People’s Hospital of Lianyungang, Lianyungang, China; 4 Institute of Nephrology, Affiliated Qingdao Third People’s Hospital, Qingdao University, Qingdao, Shandong, China; University of Utah School of Medicine, UNITED STATES OF AMERICA

## Abstract

**Aim:**

Imbalanced M1/M2 macrophage phenotype activation is a key point in diabetic kidney disease (DKD). Macrophages mainly exhibit the M1 phenotype, which contributes to inflammation and fibrosis in DKD. Studies have indicated that autophagy plays an important role in M1/M2 activation. However, the mechanism by which autophagy regulates the macrophage M1/M2 phenotype in DKD is unknown. Thus, the aim of the present study was to explore whether high glucose-induced macrophages switch to the M1 phenotype via the downregulation of STAT-3-mediated autophagy.

**Methods:**

DKD model rats were established in vivo via the intraperitoneal injection of streptozocin (STZ). The rats were sacrificed at 18 weeks for histological and molecular analysis. RAW264.7 cells were cultured in vitro with 30 mM glucose in the presence or absence of a STAT-3 activator (colivelin) and an autophagy activator (rapamycin). Moreover, M1 and M2 macrophage activation models were established as a control group. Immunofluorescence and Western blot analyses were used to detect the expression of autophagy-related proteins (LC3 and Beclin-1), M1 markers (iNOS and CD11c) and M2 markers (MR and CD206).

**Results:**

In DKD, macrophages exhibit an M1 phenotype. Under high-glucose conditions, RAW264.7 macrophages switched to the M1 phenotype. Autophagy was downregulated in high glucose–induced M1 macrophages. Both the STAT-3 activator and the autophagy activator promoted the transition of glucose-induced M1 macrophages to M2 macrophages. Moreover, STAT-3 activation increased the expression of autophagy markers (LC3 and Beclin-1). However, the autophagy activator had no effect on STAT-3 phosphorylation.

**Conclusion:**

High glucose promotes macrophage switching to the M1 phenotype via the downregulation of STAT-3-mediated autophagy.

## 1. Introduction

The progression of diabetic kidney disease (DKD) is ultimately determined by the heterogeneity of the macrophage phenotype and function [1]. Macrophages that have undergone alternative activation (M2) mediate tissue repair, whereas classically activated macrophages (M1) promote tissue inflammation and fibrosis [2]. Therefore, there is a need for more research into the regulation of the M1/M2 phenotype in macrophages.

Autophagy is one way for cells to self-degrade, providing energy for cell metabolism and maintaining cellular homeostasis [3,4]. Our previous research demonstrated that autophagy is crucial to the M1/M2 macrophage phenotype in DKD[5]. However, the molecular mechanisms that link autophagy with the macrophage phenotype in DKD remain unclear.

Signal transducer and activator of transcription 3 (STAT-3) is an important intracellular signal transduction factor that is associated with various physiological and pathological processes in cells [6,7]. STAT-3 is crucial for controlling the M1/M2 phenotype of macrophages [8,9]. However, it is unclear whether increased autophagy facilitates macrophage translation to the M2 phenotype in DKD via the upregulation of STAT-3 transcriptional activity. The purpose of the present study was to investigate how STAT-3 and autophagy affect the polarization of M1/M2 macrophages.

## 2. Materials and methods

### 2.1 Animal experiments

All animal care and experimental protocols were in compliance with the Animal Management Rules of the Ministry of Health of the People’s Republic of China. The experimental protocol was approved by the Ethical Committee of Southeast University.

Six-week-old healthy male Sprague–Dawley (SD) rats weighing 200–220 g were obtained from Shanghai Slac Laboratory Animal (Shanghai, China). After one week of acclimation, the rats were randomly divided into four groups: (1) NC (normal control group, n = 6), (2) DKD (DKD rats, n = 6). DKD was induced with a single intraperitoneal injection of STZ (Sigma, USA) dissolved in 0.1 M citrate buffer (pH 4.5) at 58 mg/kg, and the control rats received only the 0.1 M citrate buffer solution. Three days later, the diabetic state was confirmed by measuring the tail blood glucose (BG) level. Rats with a blood glucose level that greater than 16.7 mmol/L were considered diabetic rats. At the end of the experiment, the rats were executed by CO2 inhalation after anesthesia by intraperitoneal injection with tribromoethanol (Avertin, 0.2 ml/10 g body weight of a 1.2% solution, Sigma) at 18 weeks. During the treatment period, body weight was measured weekly. Blood glucose was monitored with a blood glucose monitoring system (Bayer) using one drop of tail blood. Urine samples were collected at 24 h in metabolic cages. Blood samples were taken for the measurement of biochemical parameters, and the kidneys were collected for histological examination and molecular assays.

### 2.2 Serum and urine chemistry analyses

Blood urea nitrogen (BUN) and creatinine (Scr) were analysed by an automatic biochemistry analyser (Hitachi, Tokyo, Japan). Urinary proteinuria was measured using an ELISA Kit (Jiancheng, Nanjing, China) according to the manufacturer’s method.

### 2.3 Renal histology analyses

Kidney sections were stained with periodic acid-Schiff (PAS) trichrome staining and were then examined by light microscopy (magnification, ×400) in a blinded manner. An semi-quantitative analysis of mesangial hyperplasia was evaluated in twenty randomly selected areas using the Image-Pro Plus image analysis system. The percentage of mesangial hyperplasia is the ratio of the pink mesangial area and the total glomerular area in each glomerulus.

### 2.4 Immunohistochemistry

Immunohistochemistry was performed on paraffin sections using a microwave-based antigen retrieval technique. Sections were incubated with primary mouse anti-CD68 (Santa Cruz, SC-59103), CD86 (Santa Cruz, SC-19617) followed by incubation with an appropriate secondary antibody. The immunostaining was visualized using diaminoben zidine tetrahydrochloride, and the slides were counterstained with haematoxylin.

### 2.5 Cell culture and preparation

Mouse macrophage cell line RAW264.7 was purchased from Shanghai Bogoo Biotechnology Company (Shanghai, China), were routinely cultured in RPMI 1640 media (containing 11.1 mM glucose) supplemented with 10% fetal bovine serum (Sciencell, USA) and incubated at 37°C in 5% CO_2_. RAW264.7-derived macrophages were incubated with 30mM high glucose for 24 hours. In addition, the classical activation models of M1 and M2 macrophages were established by treating cells with 100U/mL IFN-γ (Sigma, IF005) + 5 ng/mL LPS (Sigma, L5293) (M1 differentiation) for 24h or 10 ng/mL IL-4 (Sigma, SRP3211) (M2 differentiation) for 24h, respectively. Third, in order to explore the underlying mechanism, these cells were treated with or without autophagy activator Rapamycin (Rapa, Sigma, 53123-88-9), autophagy inhibitor 3-Methyladenine (3-MA, Sigma, 5142-23-4), STAT-3 siRNA (GenePharma) and STAT-3 activator Colivelin (Santa Cruz, CAS 867021-83-8).

### 2.6 Western blot

Proteins that from renal tissues or RAW264.7 cells were separated by sodium dodecyl sulfate-polyacrylamide gel electrophoresis (SDS-PAGE) and transferred to a nitrocellulose membrane. After blocking, membranes were incubated with the primary antibodies against LC3 (Abcam, ab63817), Beclin-1(Abcam, ab217179), TNF-α (Abcam, ab183218), iNOS (Abcam, ab15323), MR (Abcam, ab64693), p-STAT-3-Y705 antibody (Sigma, SAB5700314) and GAPDH at 4°C overnight. After three washes with PBST/5 min, the nitrocellulose membranes were incubated with horseradish peroxidase-conjugated secondary antibody for 1–2 h. Finally, the membranes were visualized with an enhanced chemiluminescence advanced system (GE Healthcare, UK) and captured on X-ray film. Immunoreactive bands were quantified with densitometry using Image J software (NIH, USA)

### 2.7 Electron microscopy

Cells fixed in 2% glutaraldehyde in 0.1 M potassium phosphate sodium buffer at 4°C. After postfixation with 2% osmium tetroxide, the samples were dehydrated in a series of graded ethanol solutions. Ethanol was then substituted for propylene oxide, and the samples were embedded in epoxy resin. Ultrathin sections were double stained with uranyl acetate and lead citrate. Sections were examined using a JEM1200EX electron microscope (HITACHI, Japan) at 80 keV.

### 2.8 Immunofluorescence staining

The sections of renal tissues or RAW264.7 cells were fixed and blocked. Then slides were incubated with primary antibodies: anti-CD68 antibody (Santa Cruz, sc-70761), anti-iNOS antibody (Abcam, ab15323), anti-MR antibody (Abcam, ab64693), anti-Arg-1 antibody (Sigma, ABS535), anti-p-STAT-3-Y705 antibody (Sigma, SAB5700314), anti-LC3 antibody (Sigma, L7543), CD11c (Santa Cruz, sc-398708), CD206 (Santa Cruz, sc-70585), respectively, overnight at 4°C. Then, renal tissues or cells were washed and incubated with appropriate secondary antibody in darkness for 2h at 37°C. After staining nuclei with DAPI, renal tissues or cells were visualized using an IX70 fluorescence microscope (Olympus, Japan).

### 2.9 Statistical analysis

All experiments were repeated at least three times. The data are expressed as the mean and the standard deviation (SD) and were analyzed with SPSS 19.0. One-way ANOVA was used for multiple groups, SNK test was adopted for two comparisons, and the correlation analysis between the two variables was analyzed by Pearson correlation. A difference was considered significant if the *P* value was less than 0.05.

## 3. Results

### 3.1 Association of autophagy, STAT-3, and the macrophage phenotype in STZ-induced rat kidneys

**Fig 1:**
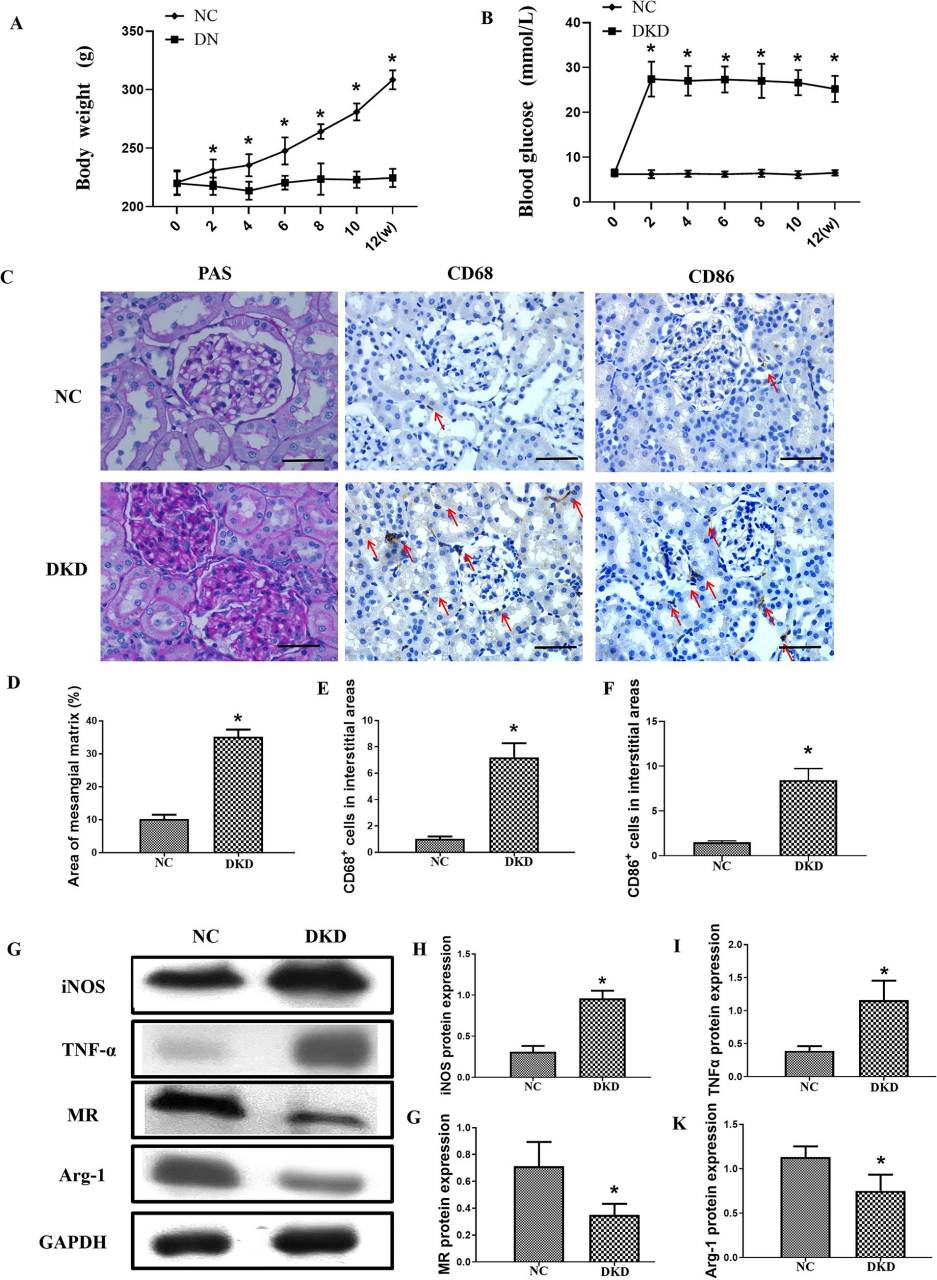
Histopathological features and expression of macrophage phenotype markers, autophagy markers, and p-STAT-3 in the kidneys of DKD rats. A, Body weight; B, Blood glucose; C, PAS stains of patients’ renal tissues (×400, bar = 20μm); Immunohistochemical staining of CD68 and CD86 macrophages in the interstitial areas (×400, bar = 50μm). D—F: Semiquantitative analysis of the glomerular mesangial matrix and quantification of the number of CD68 and CD86 positive macrophages per interstitial area. G—K, representative Western blotting analysis and quantification of M1 (iNOS and TNF-α) and M2 (MR and Arg-1) makers. The data are presented as the mean ± SD (n = 6 per group). *P<0.05 vs NC (normal control) group. MR: Mannose Receptor; Arg-1: Arginase 1.

At the beginning of the study, there was no difference in blood glucose or body weight between the two groups. Compared with the NC rats, the DKD rats developed overt diabetes after STZ injection, with lower body weights and higher blood glucose levels (P<0.05, Fig 1A and 1B). As expected, the DKD rats presented increased proteinuria (P<0.05). Table 1 shows the baseline and final parameters of the DKD rats. Scr, BUN, and kidney-to-body weight ratios all increased significantly in DKD rats (Table 1). The kidneys of DKD rats presented severe morphological lesions. In DKD rats, the glomerular surface area increased, and the glomerular mesangial matrix extended (Fig 1C). According to the immunohistochemistry results, the DKD group had significantly more CD68-positive and CD86-positive macrophages. (P<0.05, Fig 1E and 1F).

**Table 1:** The general parameters in experimental animals at 18 weeks.

	Proteinuria (mg/24h)	KW/BW (mg/g)	Scr (μmol/L)	BUN (mmol/L)
NC	9.65±1.92	3.04±0.37	32.01±8.34	5.93±0.88
DKD	58.74±6.13 *	6.56±0.82 *	86.52±9.95 *	16.61±2.37 *

Table 1. Parameters of the experimental groups of rats.

KW/BW: Kidney weight/body weight; Scr: Serum creatinine; BUN: Blood urea nitrogen. Date are presented as mean±SD (n = 6).

*P<0.05 vs NC.

Western blot analysis was used to determine the protein levels of CD68, M1 markers (iNOS and TNF-α), and M2 markers (MR and Arg-1) in the renal tissues of STZ-induced DKD rats. The DKD group presented higher levels of CD68 and M1 marker expression compared to the NC group. In contrast, the expression of the MR M2 marker was lower in the treated group than in the NC group (P<0.05, Fig 1G–1K).

**Fig 2:**
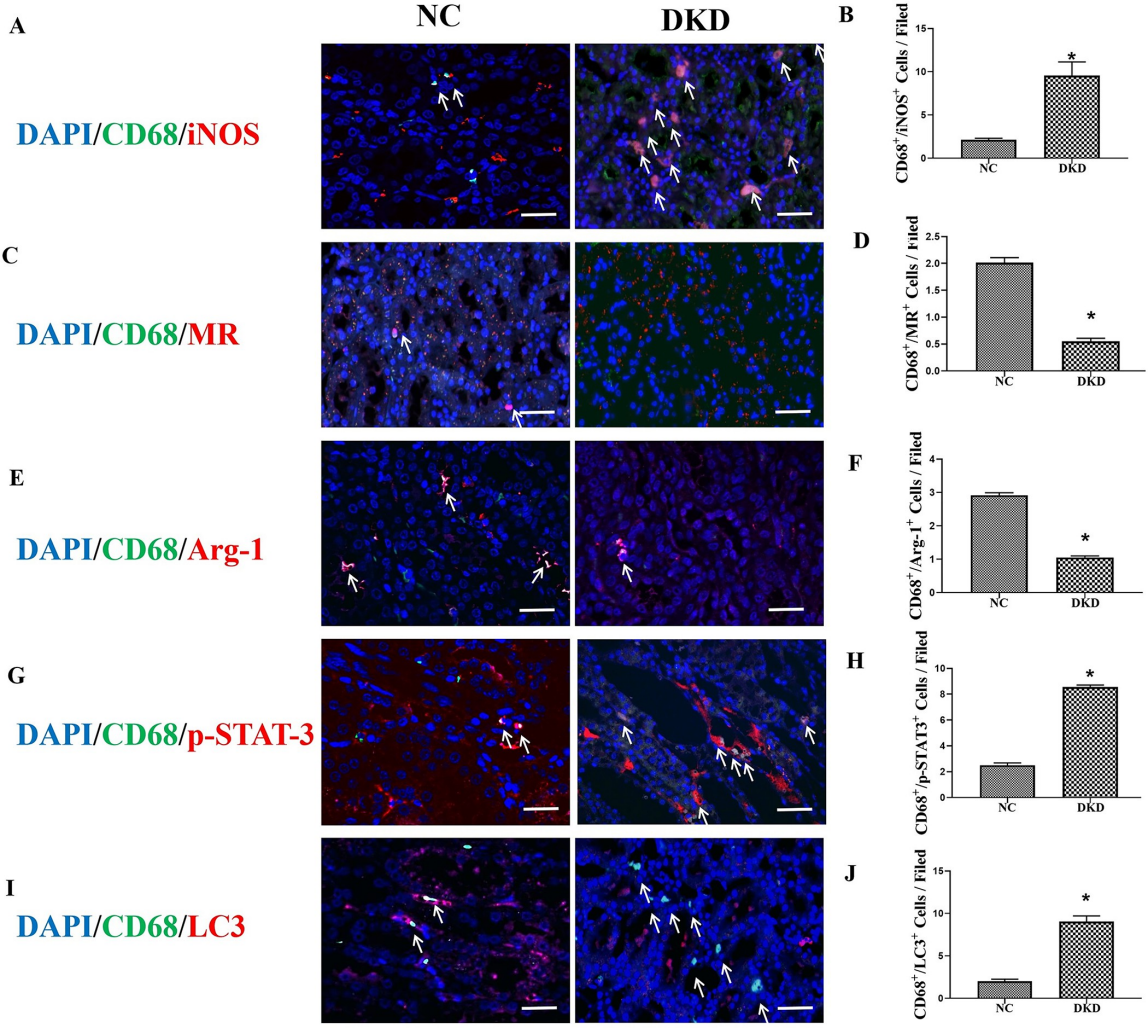
Coexpression of macrophages and the LC3 and p-STAT-3 macrophage phenotype markers in the kidneys of DKD rats. Double-immunofluorescence images and quantification of CD68 and iNOS (A and B), CD68 and MR (C and D), CD68 and Arg-1 (E and F), CD68 and p-STAT-3 (G and H), CD68 and LC3 (I and J) were detection in rat kidney tissue (white arrows) (×400; bars = 50μm). MR: Mannose Receptor; Arg-1: Arginase 1.

Immunolabeling and confocal microscopy imaging of CD68, iNOS, MR, Arg-1, p-STAT-3, and LC3 in rat kidney tissue were performed. In DKD rats, CD68 and iNOS colocalization was increased, whereas MR or Arg-1 and CD68 colocalization was decreased (Fig 2A–2F). The colocalization of CD68 with LC3 and p-STAT-3 was confirmed by confocal immunofluorescence (Fig 2G–2J).

### 3.2 High glucose affects autophagy, STAT-3, and the macrophage phenotype in vitro

**Fig 3:**
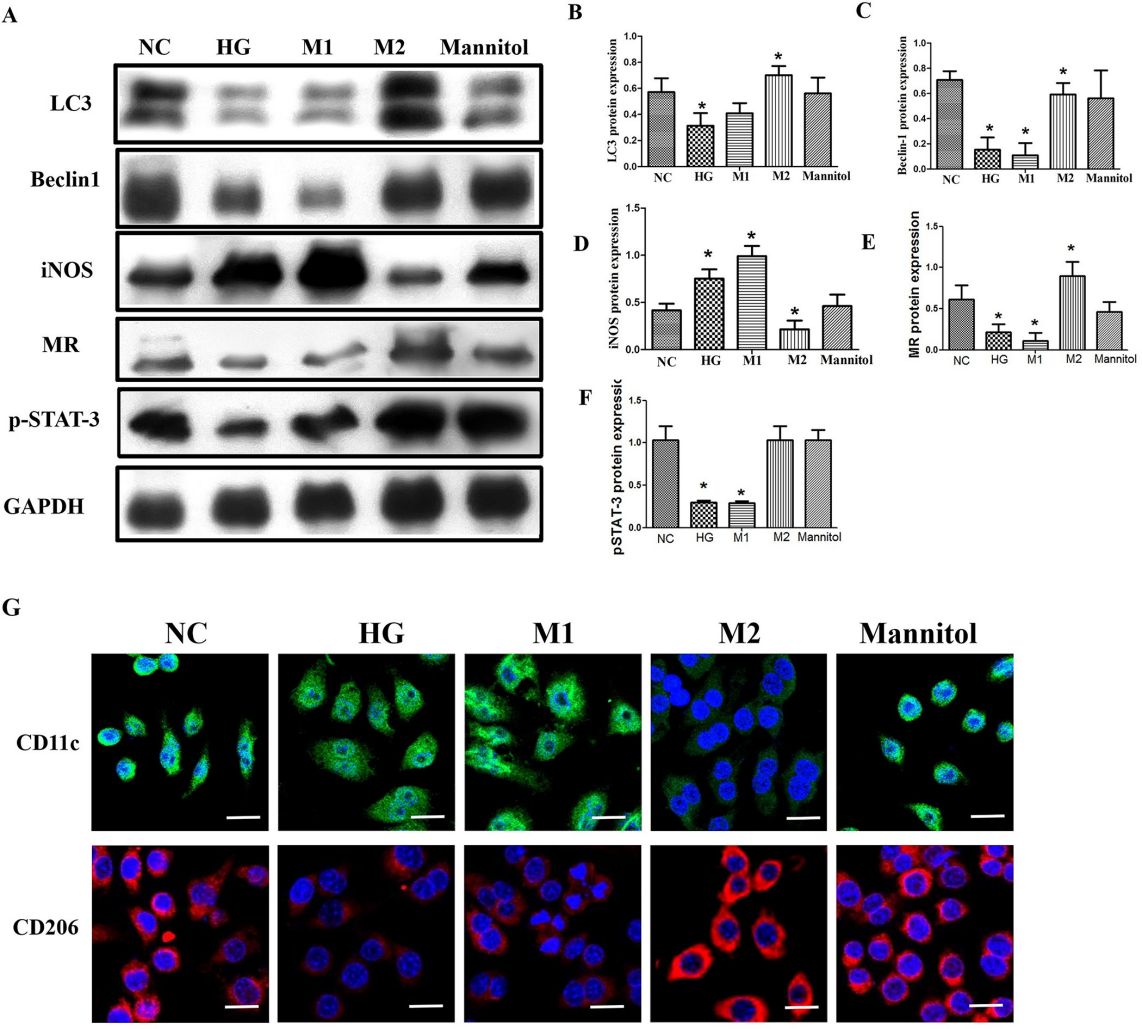
Effects of high glucose on macrophage phenotype, autophagy, and p-STAT-3 in RAW264.7 cells. A-F, Representative Western blotting analysis and quantification of LC3, Beclin-1 (C), iNOS, MR, p-STAT-3. GAPDH was used as an internal control. Data are presented as mean± SD (n = 3). *P<0.05 vs NC (normal control), respectively. G, the immunofluorescence analysis of HG (high glucose) on macrophage phenotype (×400; bars = 20μm). NC:11mmol/L glucose, HG: 30 mmol/L glucose; Mannitol: 11mmol/L glucose + 19 mmol/L mannitol. MR: Mannose Receptor.

To determine whether high glucose induces autophagy changes and macrophage phenotype transformation, several specific markers of macrophage phenotype and autophagy were examined in protein extracted from RAW264.7 cells via western blot analysis and immunofluorescence staining. In addition, as positive controls, classical activation models of M1 and M2 macrophages were established. Compared with those in the normal control group, the expression levels of autophagy markers (LC3 and Beclin-1), M2 markers (MR), and p-STAT-3 decreased, whereas the expression levels of M1 markers (iNOS) increased (Fig 3). Furthermore, the protein and fluorescence expression patterns were parallel to those of classical M1 macrophage activation models.

### 3.3 Both autophagy and the M1/M2 macrophage phenotype are regulated by STAT-3 activators

To explore whether STAT-3 regulates the high glucose-induced macrophage phenotype and autophagy, western blot analysis and immunofluorescence staining were used to examine the expression of macrophage phenotype and autophagy marker proteins in RAW264.7 cells.

**Fig 4:**
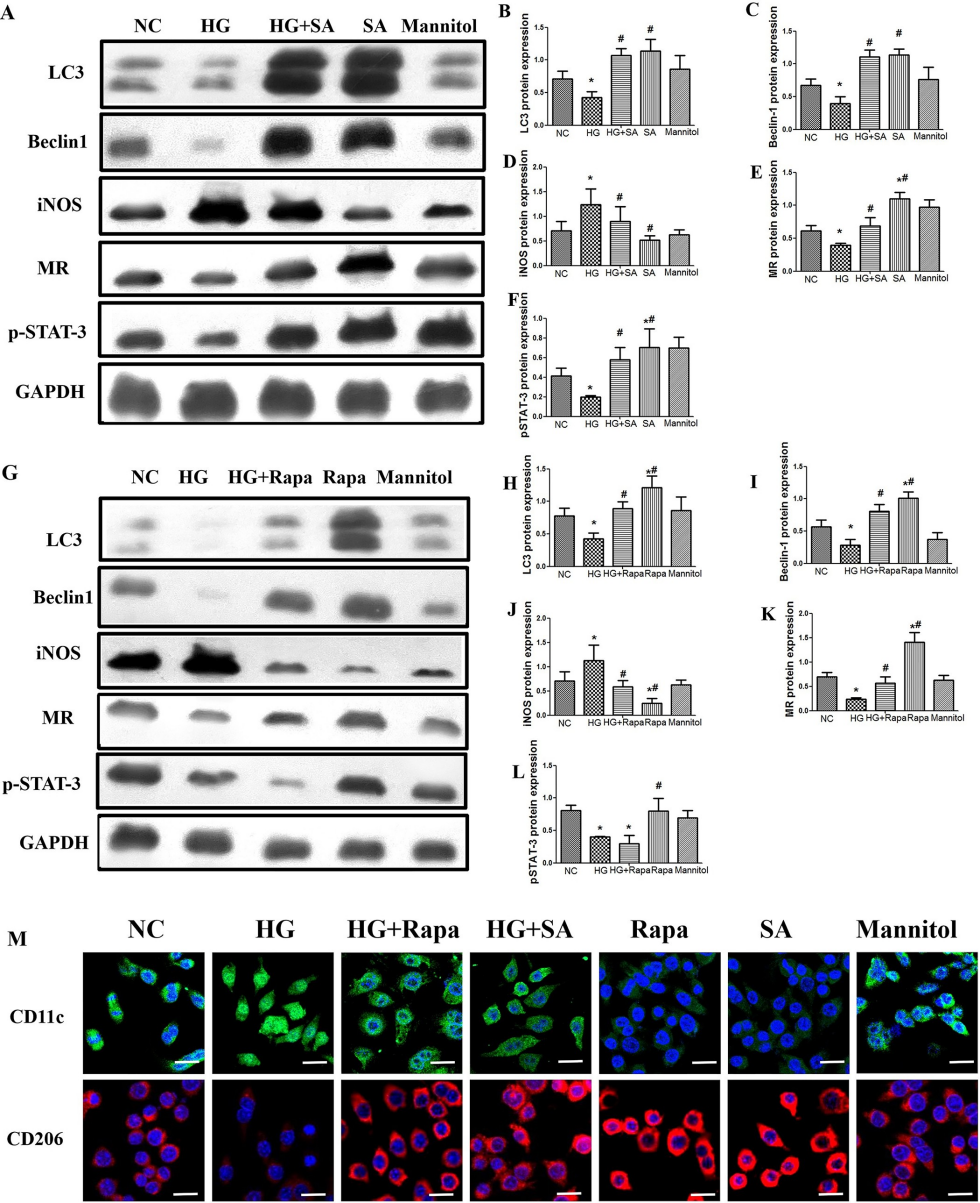
Effects of STAT-3 activators and autophagy activators on macrophage phenotype, autophagy, and STAT-3 expression in RAW264.7 cells. L, Representative Western blotting analysis and quantification (B) of LC3, Beclin-1, iNOS, MR, p-STAT-3. GAPDH was used as an internal control. Data are presented as mean ± SD (n = 3). M, the immunofluorescence analysis of STAT-3 activator and autophagy activator (Rapamycin, Rapa) on macrophage phenotype (×400; bars = 20μm). *P<0.05 vs NC (normal control), ^#^P<0.05 vs HG. NC:11mmol/L glucose, HG: 30 mmol/L glucose; Mannitol: 11mmol/L glucose + 19 mmol/L mannitol. MR: Mannose Receptor; SA: STAT-3 activator, Rapa: Rapamycin.

The cells were first divided into several groups at random. Following STAT-3 activator treatment, the expression of autophagy markers (LC3 and Beclin-1) and macrophage phenotypic markers (iNOS and MR) was observed under different conditions. The STAT-3 activator promoted the transformation of M1 macrophages induced by high glucose to the M2 phenotype and increased autophagy. Under high-glucose conditions, the STAT-3 activator promoted high expression of an M2 marker (MR) and autophagy markers (LC3 and Beclin-1), while it downregulated the expression of an M1 marker (iNOS) (Fig 4).

### 3.4 Autophagy activators are essential for the regulation of the M1/M2 macrophage phenotype but not p-STAT-3 expression

The present study next determined whether STAT-3 and the high glucose-induced macrophage phenotype are regulated by autophagy. First, the concentration of the autophagy activator (rapamycin) was selected based on a previous study [5]. RAW264.7 cells were subsequently grown for 24 h with 30 mM glucose with or without the addition of rapamycin. Western blot analysis was used to measure the expression of the autophagy-related proteins (LC3 and Beclin-1), the M1 marker (iNOS), and the M2 marker (MR) (Fig 4). The M1 (CD11c) and M2 (CD206) macrophage phenotypes were further determined by immunofluorescence staining. Rapamycin significantly increased M2 marker (MR and CD206) expression but decreased M1 marker (iNOS and CD11c) expression. Moreover, rapamycin did not affect the expression of p-STAT-3, which was inhibited by high glucose levels.

**Fig 5:**
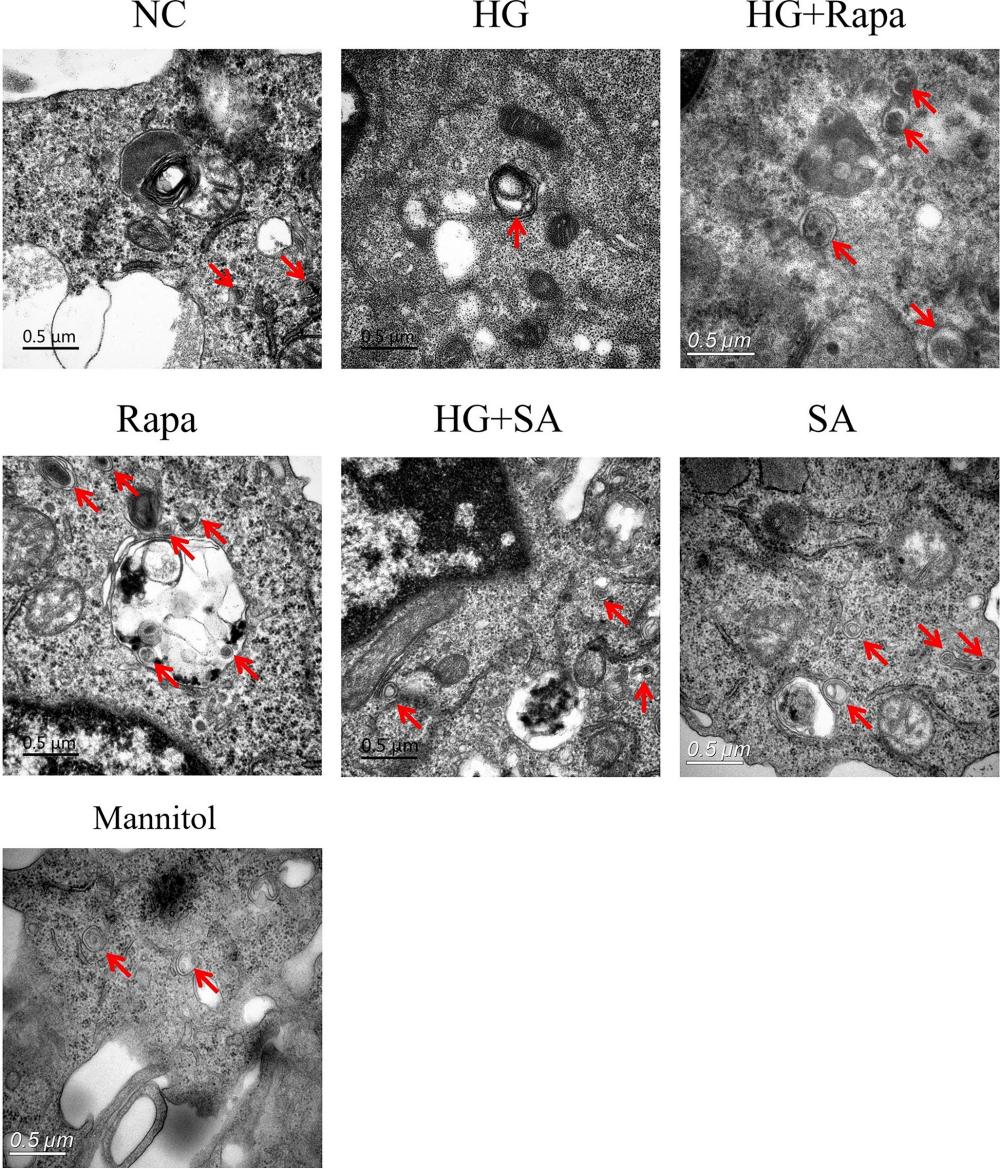
Autophagy in RAW264.7 cells. The numbers of autophagosomes were decreased in HG group compared with NC group, but increased after STAT-3 activator (SA) and autophagy activator (Rapamycin, Rapa) treatment. Scale bar indicates 0.5μm. Red arrow: autophagosome. NC:11mmol/L glucose, HG: 30 mmol/L glucose; Mannitol: 11mmol/L glucose + 19 mmol/L mannitol.

The numbers of autophagosomes in RAW264.7 cells were subsequently compared via electron microscopy (Fig 5). Compared with the NC group, the number of autophagosomes in the high-glucose group was decreased, but the number of autophagosomes in the STAT-3 activator and autophagy activator (rapamycin) treatment groups was increased.

**Fig 6:**
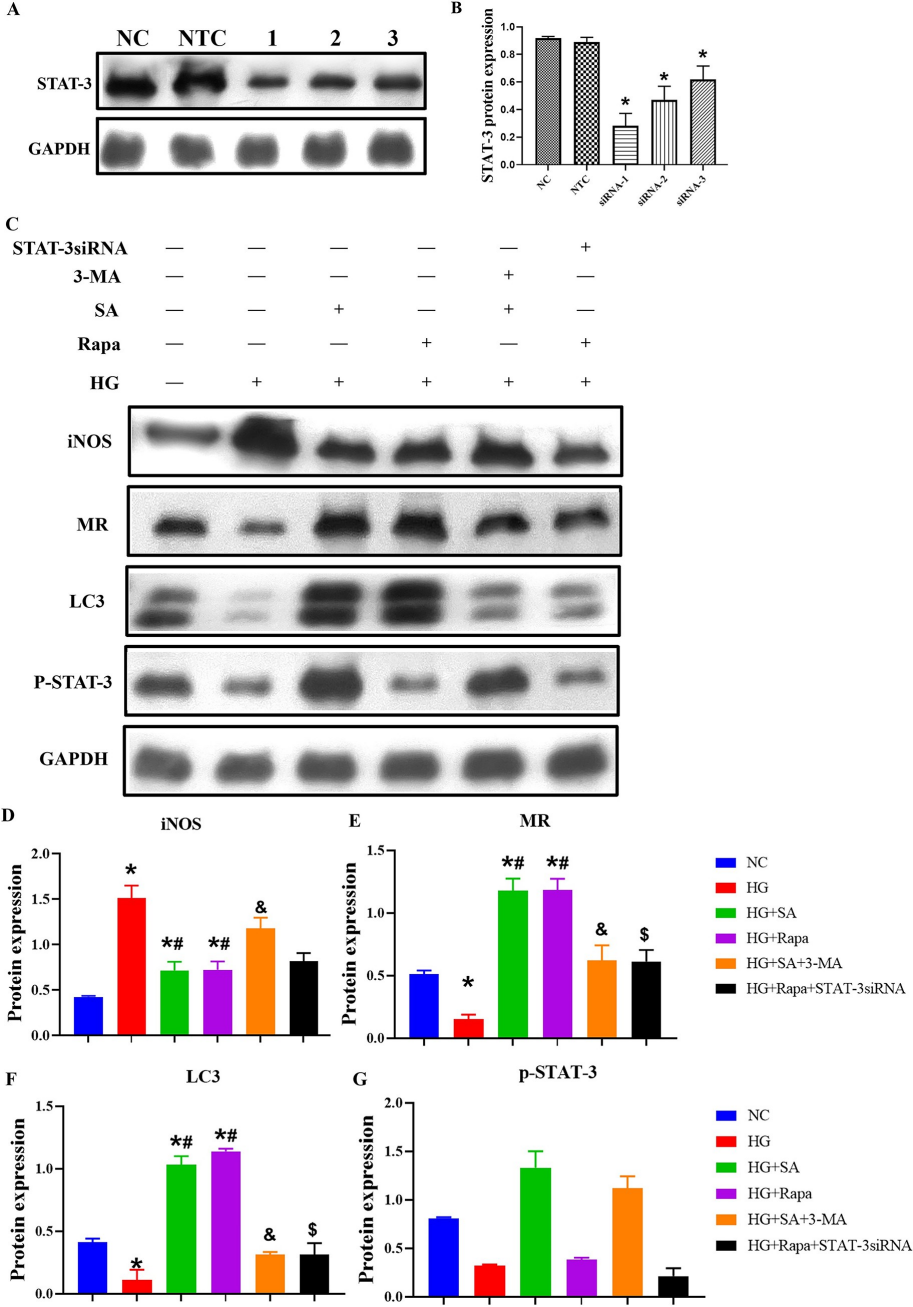
Protein expression of p‐STAT‐3, LC3, iNOS, and MR under different conditions. A and B, The effect of STAT-3 siRNA on STAT-3 expression. *p < 0.05 vs. NC group; NC: Normal control; siRNA: Small interfering RNA; NTC: Nontarget control (NTC) siRNA. C-G: RAW264.7 cells were treated with different reagents. The cells were collected for western blot analysis, quantification analysis of p-STAT-3, LC3, iNOS and MR. data are presented as the mean ± SD (n = 3 per group). *p < 0.05 vs. NC group; #p < 0.05 vs. HG group, &p < 0.05 vs. HG+SA group, $p < 0.05 vs. HG+Rapa group. HG: High glucose; MR: Mannose receptor; STAT: Signal transducer and activator of transcription; Rapa: Rapamycin; SA: STAT-3 activator; 3-MA:3-Methyladenine. NC:11mmol/L glucose, HG: 30 mmol/L glucose; Mannitol: 11mmol/L glucose + 19 mmol/L mannitol.

To further clarify the effect of STAT‐3-mediated autophagy, siRNAs targeting STAT‐3 were transfected into RAW264.7 cells, and an NTC siRNA was used to control for the nonspecific effects of the transfection reagents. The inhibition ratios of STAT‐3 siRNA‐1, siRNA-2, and siRNA-3 were 69.7%, 45.5%, and 22.9%, respectively; therefore, STAT‐3 siRNA‐1 was used as the final intervention siRNA (Fig 6A and 6B). The expression of iNOS, MR, p-STAT-3, and LC3 was evaluated via western blot analysis in the HG+SA (STAT-3 activator)+3MA (autophagy inhibitor) and HG+Rapa (autophagy activator plus rapamycin) +STAT-3 siRNA groups. High glucose induced M1 marker (iNOS) expression, but the effects of high glucose were abolished when autophagy and STAT‐3 were activated. A reduction in STAT‐3 expression by STAT-3 siRNA decreased autophagy, whereas the regulation of autophagy caused no obvious changes in p‐STAT‐3 expression (Fig 6).

## 4. Discussion

Macrophage function is divided into two phenotypes. The activation of the M1/M2 phenotype of macrophages determines the progression and prognosis of renal injury [10]. Our team has focused on investigating the macrophage phenotype and how it affects the prognosis of renal diseases in recent years. Our previous research demonstrated that the renal tissue of STZ-induced DKD model rats primarily includes M1 macrophages, with a smaller proportion of M2 macrophages [11]. Under high-glucose conditions in vitro, RAW264.7 macrophages tend to transition to the M1 phenotype and express increased levels of proinflammatory cytokines [11–13]. We further revealed that human renal tissue from DKD patients is infiltrated by macrophages with two distinct phenotypes, namely, M1 and M2. There is an imbalance in the M1 and M2 phenotypes of macrophages in DKD renal tissue [14]. In certain circumstances, the M1 and M2 phenotypes undergo reciprocal transformations, in which each plays a different role in injury or repair. However, the mechanism of M1/M2 macrophage phenotypic modulation remains unexplained.

An intracellular degradation system, known as autophagy, is linked to the maintenance of cellular homeostasis. Recently, many studies have shown that autophagy and DKD are closely connected. Research has indicated that renal tissue from DKD model rats has decreased expression of autophagy markers [15–17]. Wu [18] reported that under high-glucose conditions, podocyte cells undergo less autophagy. The present study demonstrated that the expression of p-STAT-3 and autophagy level were decreased under HG conditions. The present study further investigated the impact of autophagy on the M1/M2 classical model, demonstrating that the level of autophagy in M2 macrophages was substantially greater than that in M1 macrophages. Moreover, autophagy markers and the M1 phenotype of macrophages were positively associated with p-STAT-3. More research is necessary to determine whether modulating autophagy and p-STAT-3 can modify macrophage phenotypes.

Autophagy is tightly linked to the regulation of macrophage function. In the regulation of macrophage activity, autophagy is essential [19–22]. Duan discovered that enhancing the autophagy response efficiently protects macrophages from stress-induced cytotoxicity and enhances their phagocytic capacity [23]. In db/db mice, Guo discovered that a regulator of autophagy activation upregulates autophagy, impairs wound healing, and enhances the inflammatory response [16]. These investigations have revealed that macrophage activity is regulated mostly by autophagy. We recently reported that regulating autophagy impacts the phenotypic transformation of M1/M2 macrophages in DKD rats [5]. The present study demonstrated that autophagy activator stimulation downregulated high glucose-induced overexpression of iNOS but significantly upregulated M2 markers (MR) and autophagy markers (LC3 and Beclin1). In addition, the expression of these markers paralleled that of IL-4-induced M2 macrophage activation. The rapamycin autophagy activator significantly reduced the activation of M1 macrophages, and it increased the activation of M2 macrophages; specifically, it increased the expression of an M2 marker (MR) but decreased the expression of an M1 marker (iNOS). However, rapamycin had no effect on p-STAT-3 expression.

Signal transducer and activator of transcription 3 (STAT-3) is a key intracellular signal transduction factor that controls the maturation and differentiation of various immune cells [7,24]. Gordon et al. suggested that STAT-3 is primarily responsible for the activation of M2 macrophages; they reported that IL-10 is blind to its receptor and activates STAT-3 and the transcription of M2-related genes, including Arg-1, thereby promoting anti-inflammatory and immunomodulatory effects [25]. In mice with ischemia-induced liver injury, Martinez reported that STAT1 and STAT-3 signal balance problems are closely associated with the M1/M2 macrophage phenotype [26]. The present study revealed in high-glucose environments, RAW264.7 macrophages shifted to the M1 phenotype and had lower autophagy levels. Furthermore, the expression of macrophage phenotypes and autophagy biomarkers paralleled that of LPS- and IFN-induced M1 macrophage activation.

In conclusion, the present study is the first to explore the influence of the STAT-3-autophagy interaction on the M1/M2 phenotype under high-glucose conditions. High glucose levels promote the switch of M1 macrophages to M2 macrophages by inhibiting STAT-3-mediated autophagy. However, the mechanism through which STAT-3 regulates autophagy is unclear. Therefore, further clarification of the regulatory mechanism between STAT-3 and autophagy will provide an experimental framework and suggestions for regulating the M1/M2 phenotype in macrophages.

## Supporting information

S1 Raw data Data presented in the figures. (XLSX)

S1 File Raw images. The raw images of Western blot.
(PDF)
