# Supplementary material for: High glucose promotes macrophage switching to the M1 phenotype via the downregulation of STAT-3 mediated autophagy
Source: PLoS One. 2024 Dec 31;19(12):e0314974. doi: 10.1371/journal.pone.0314974 (PMC11687880; doi:10.1371/journal.pone.0314974)
Supplement: S1 File — The raw images of Western blot. (PDF) [file pone.0314974.s002.pdf]

Fig.1

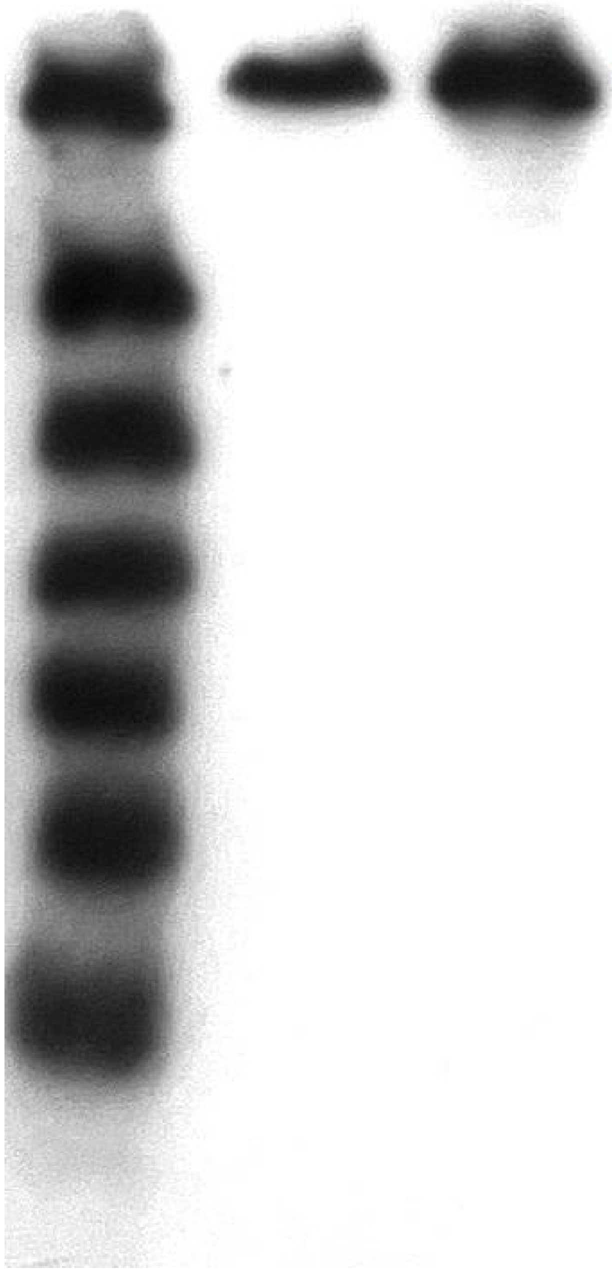

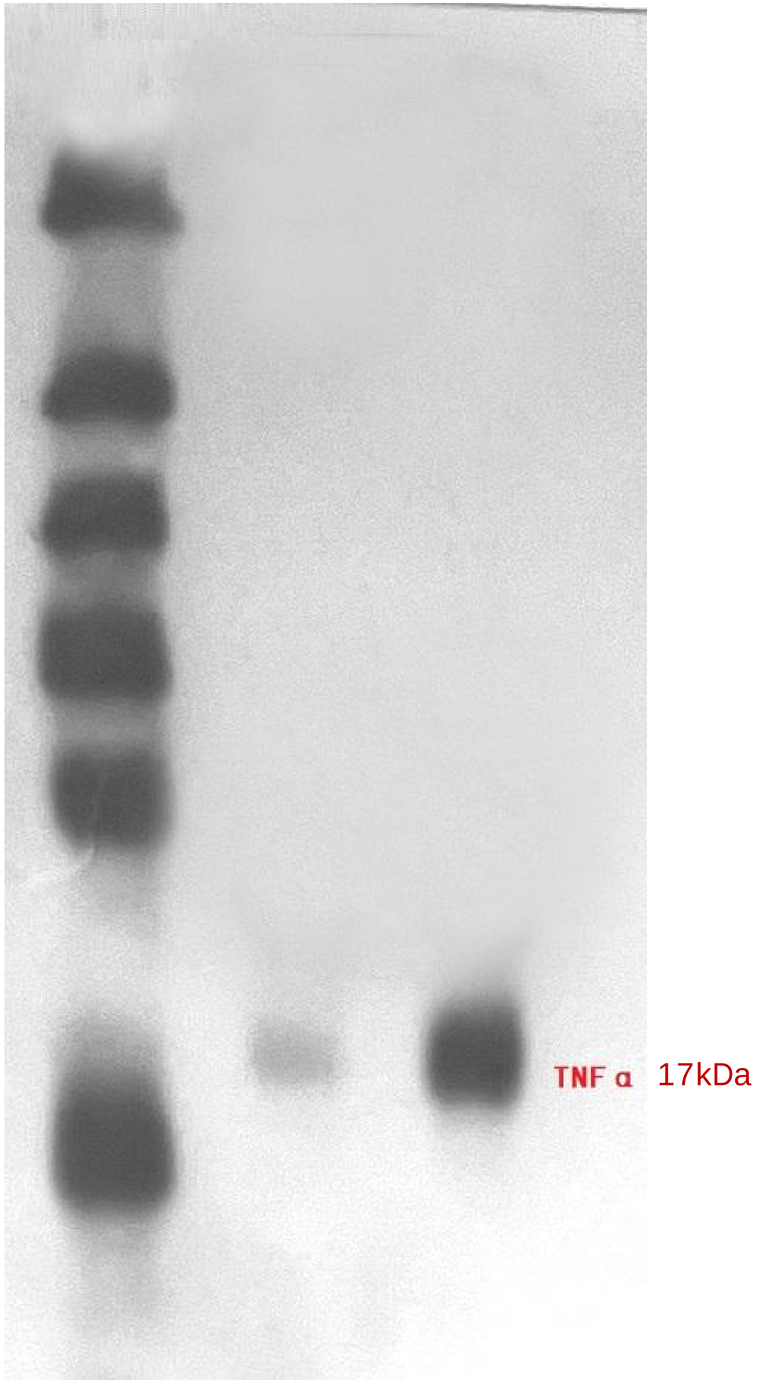
TNF α 17kDa

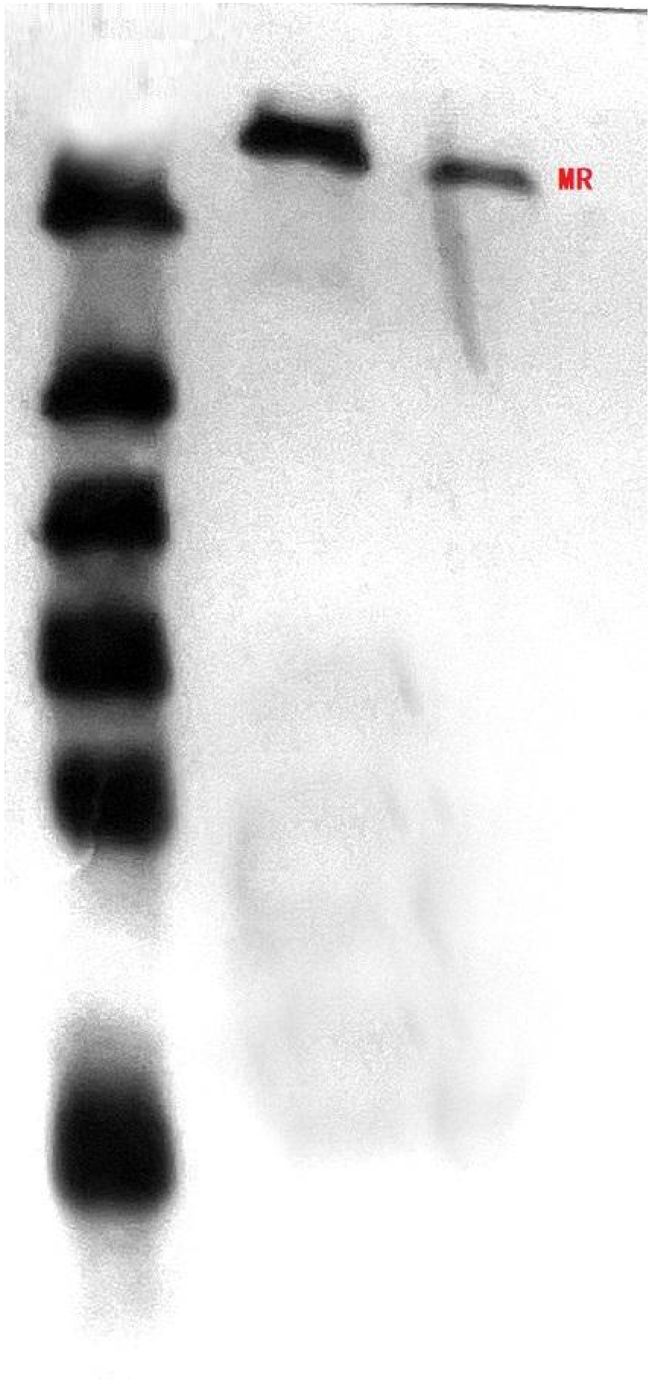

34kDa

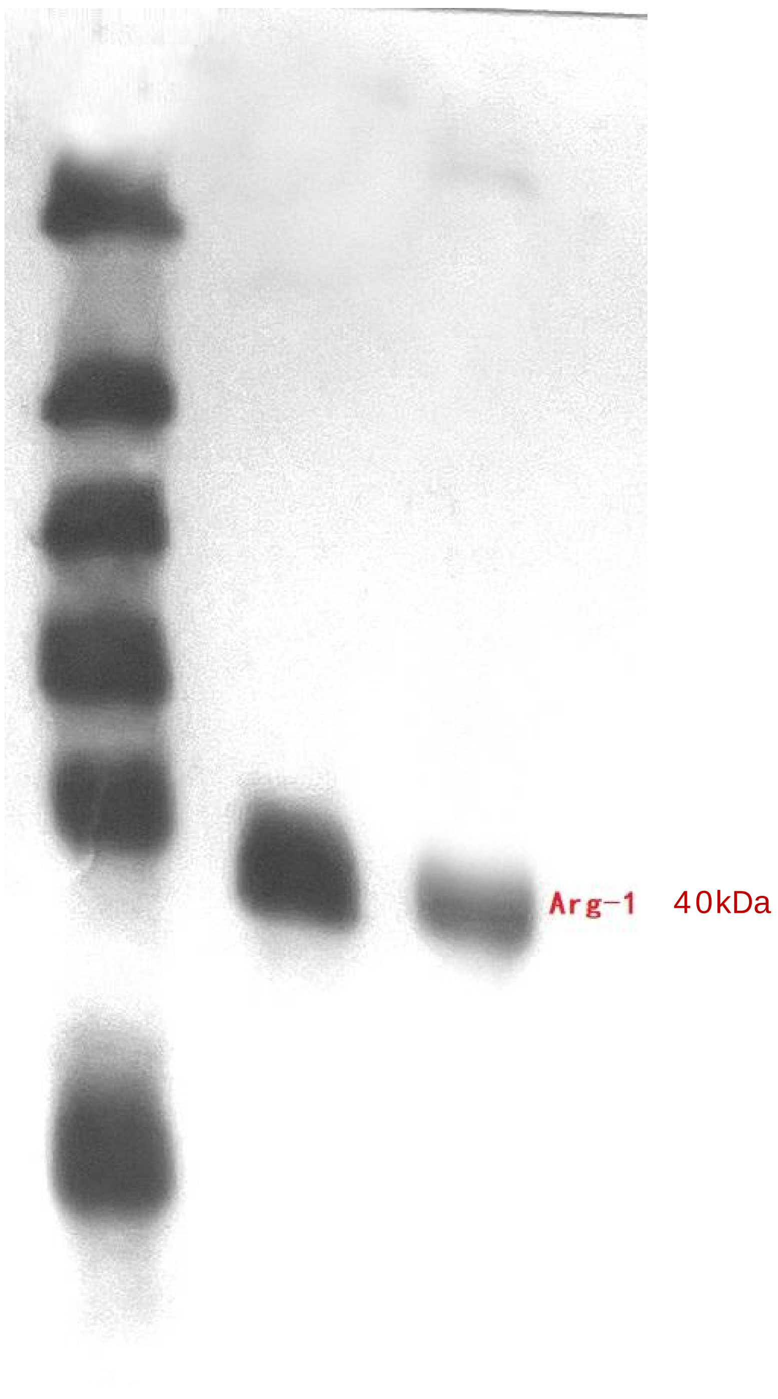
Arg-1
40kDa

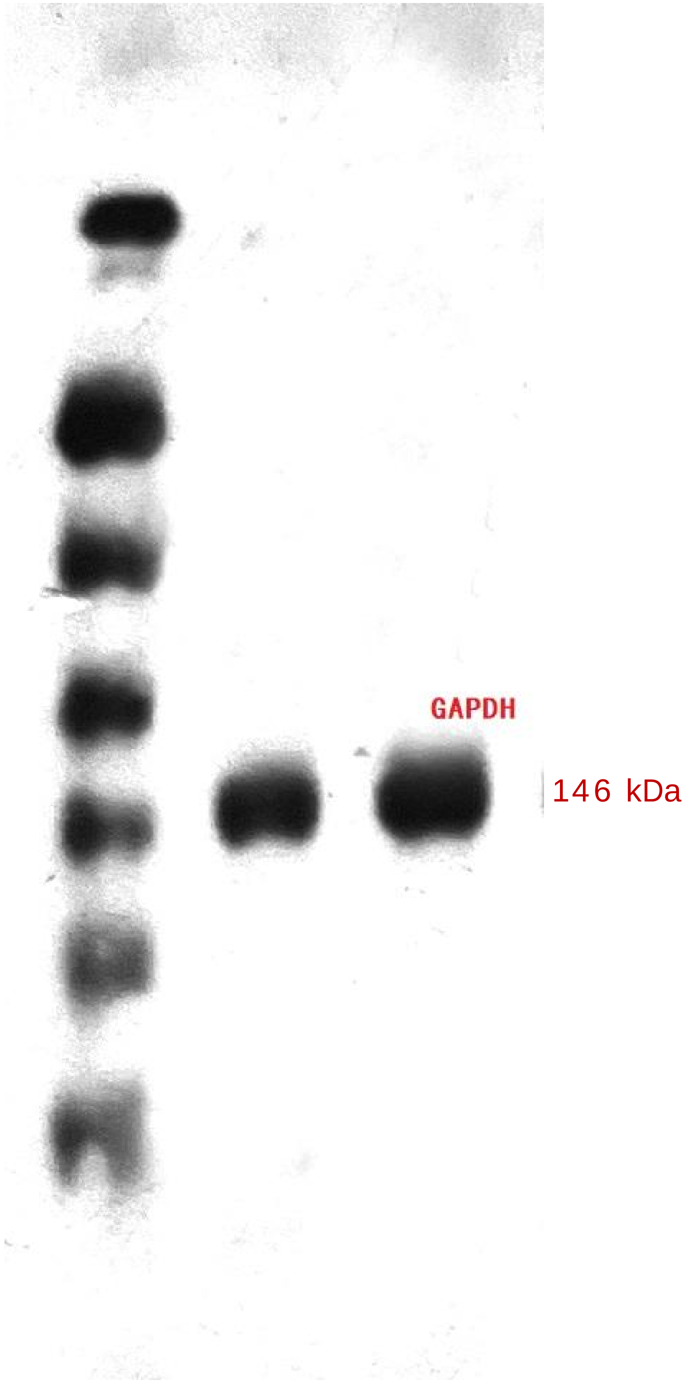
GAPDH
146 kDa

Fig.3

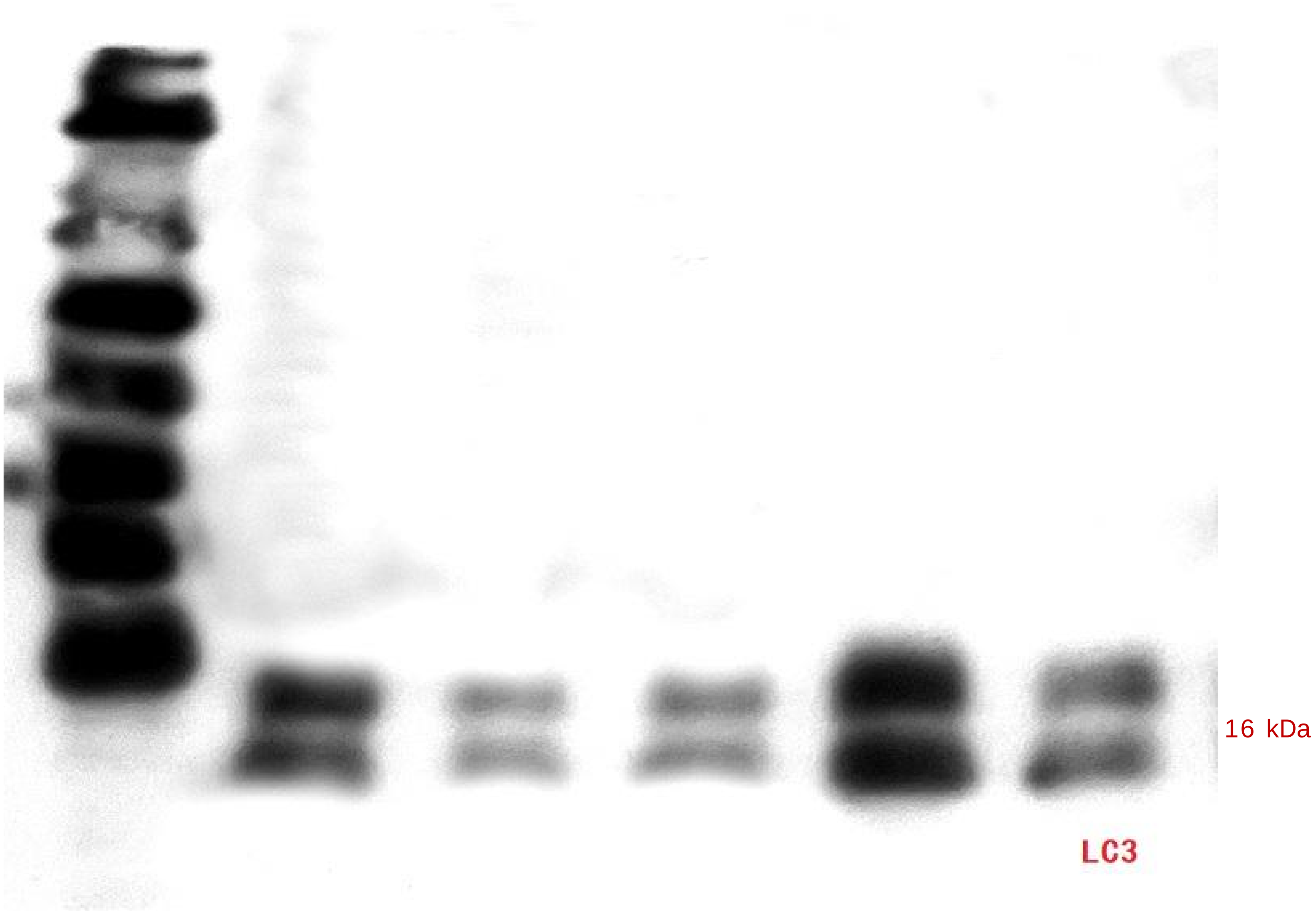

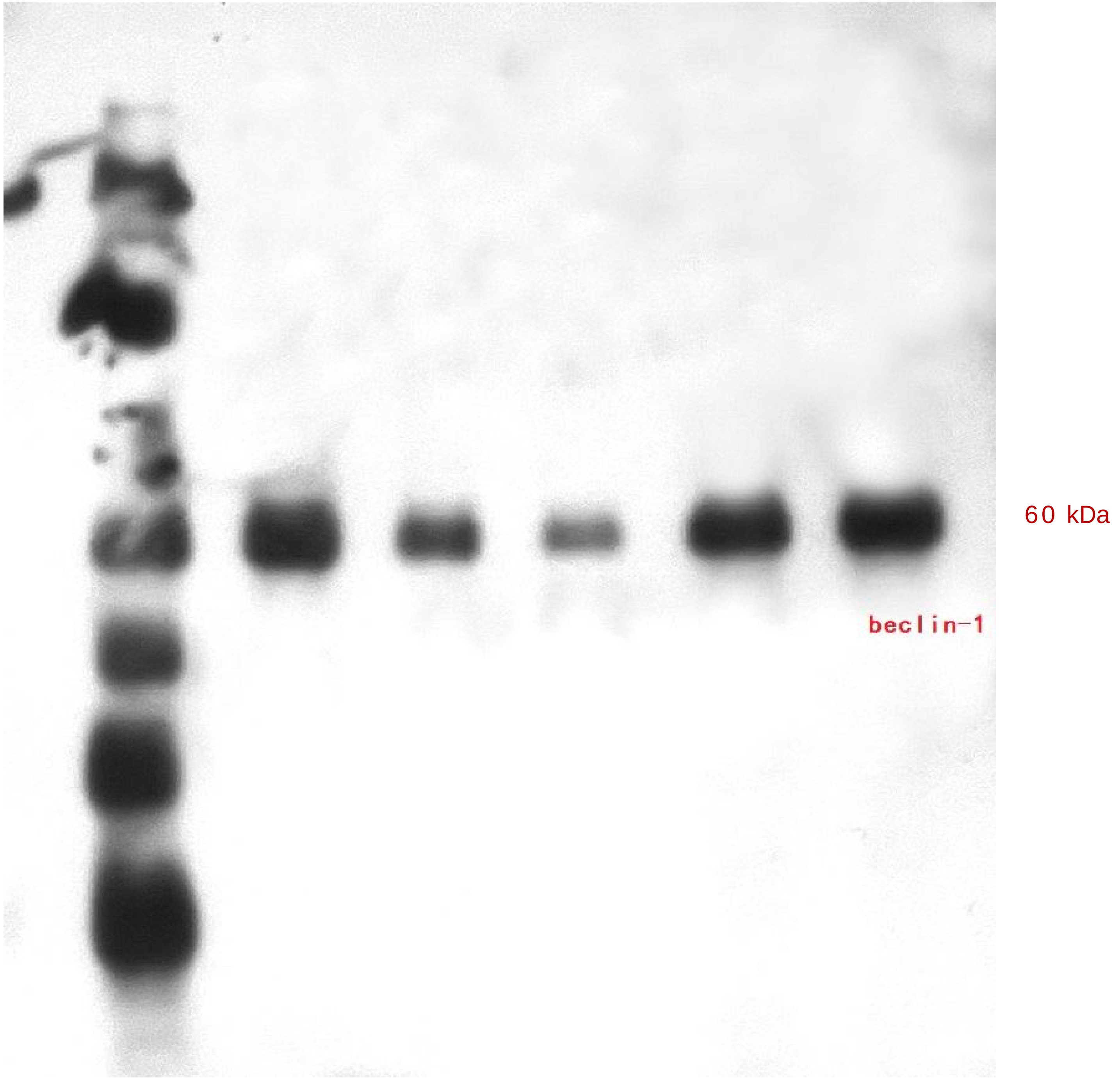
60 kDa
beclin-1

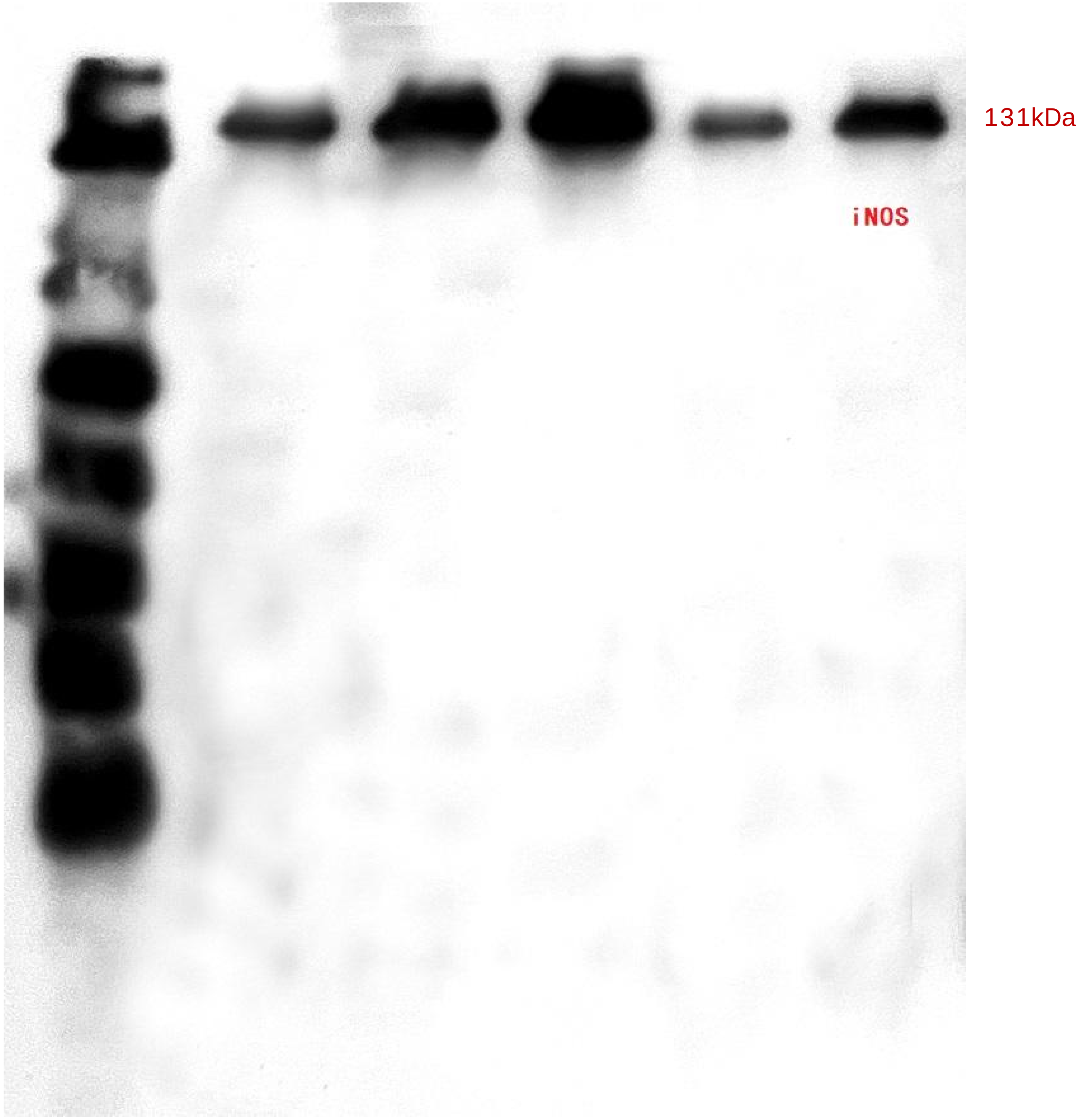
131kDa
iNOS

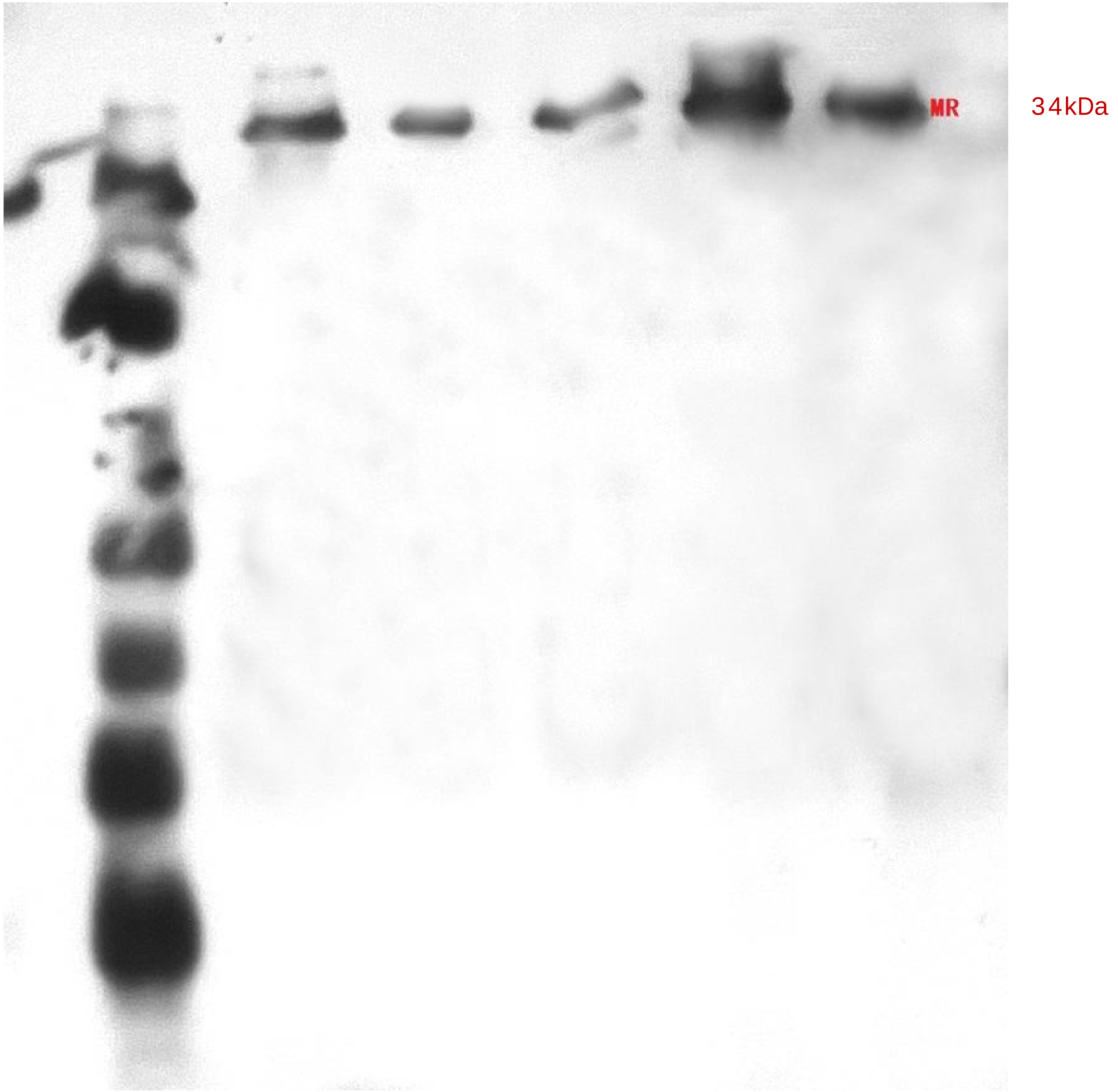
MR
34kDa

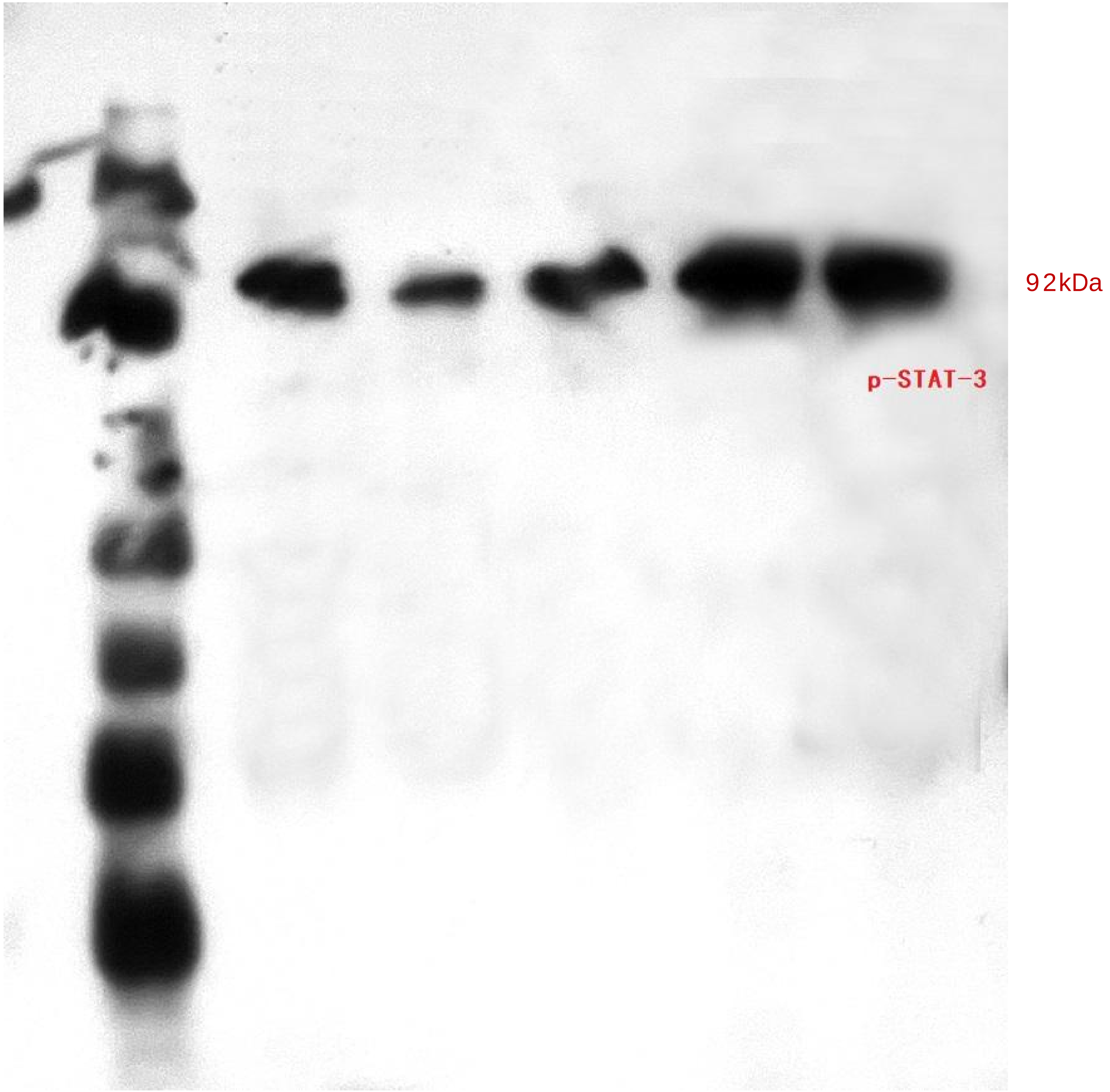
92kDa
p-STAT-3

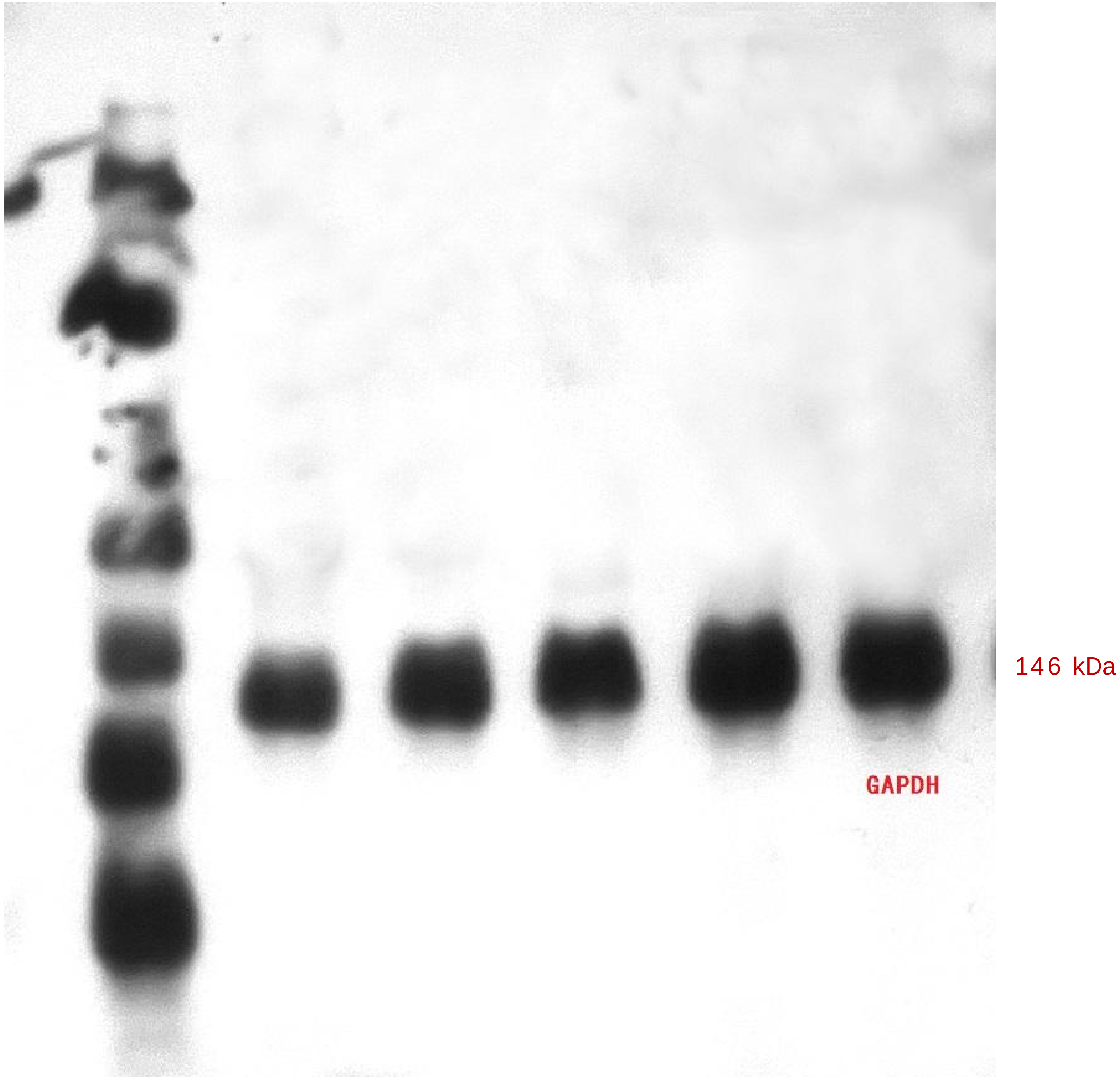
146 kDa
GAPDH

Fig. 4A

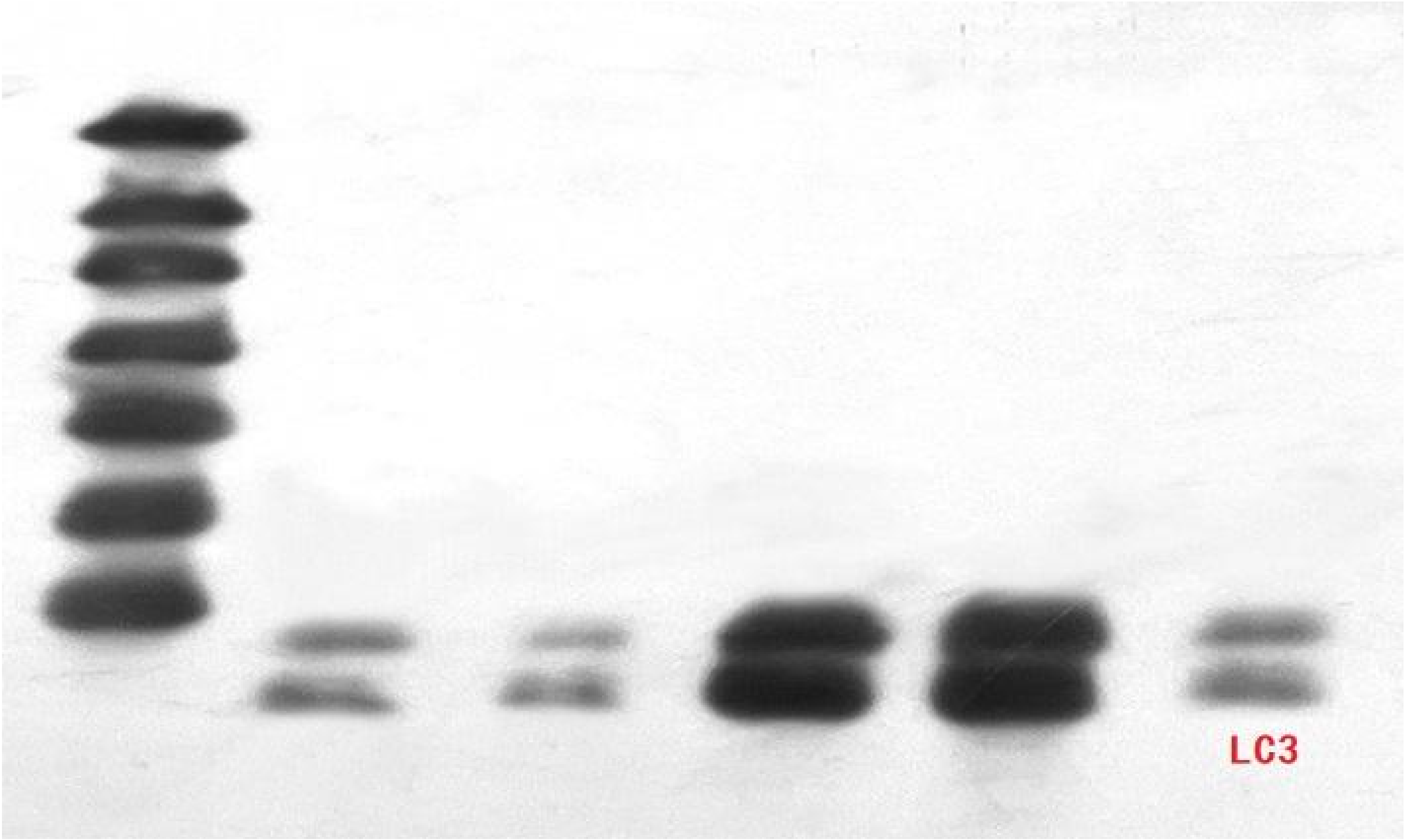

16 kDa

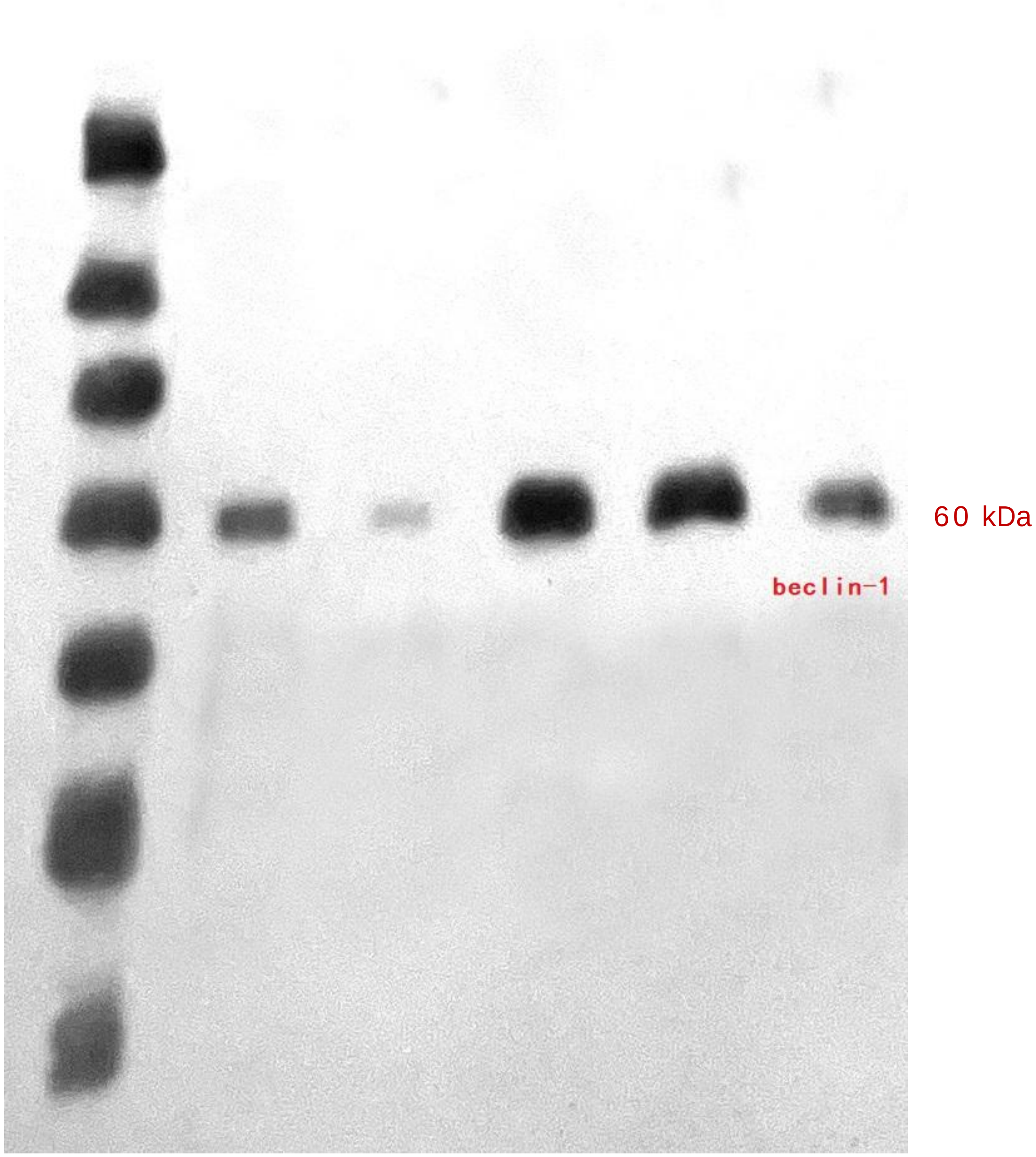
60 kDa
beclin-1

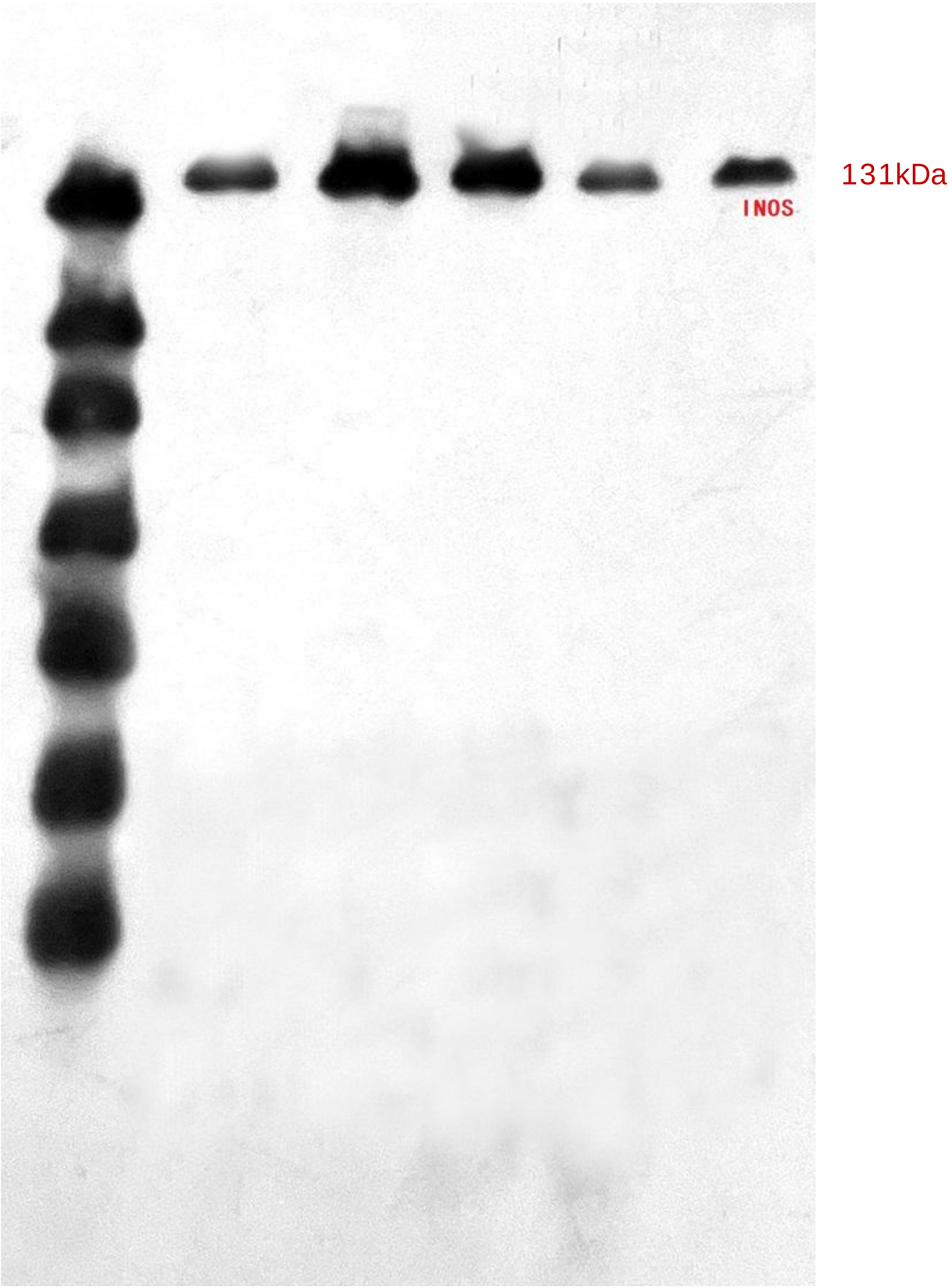
131kDa
INOS

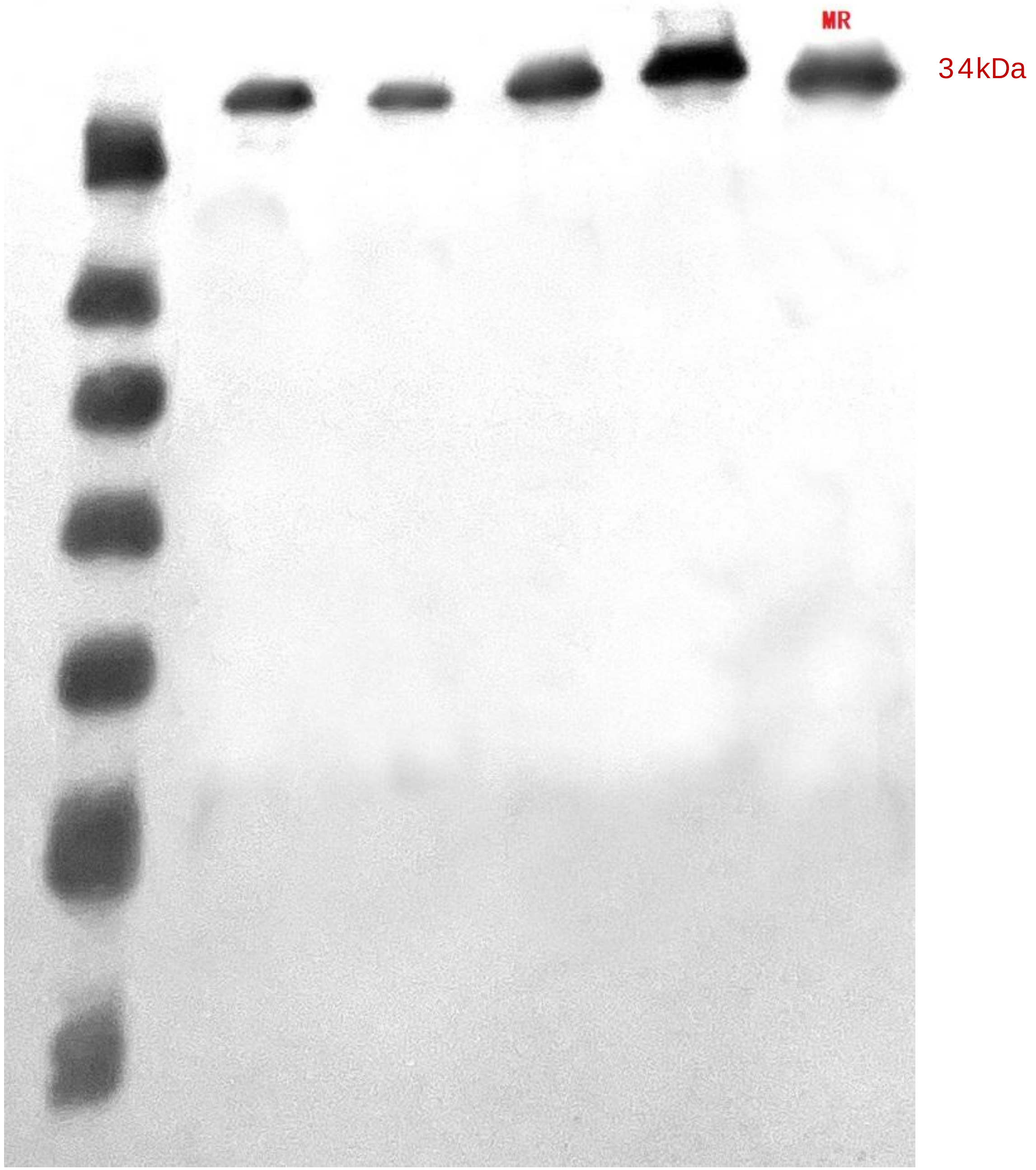
MR
34kDa

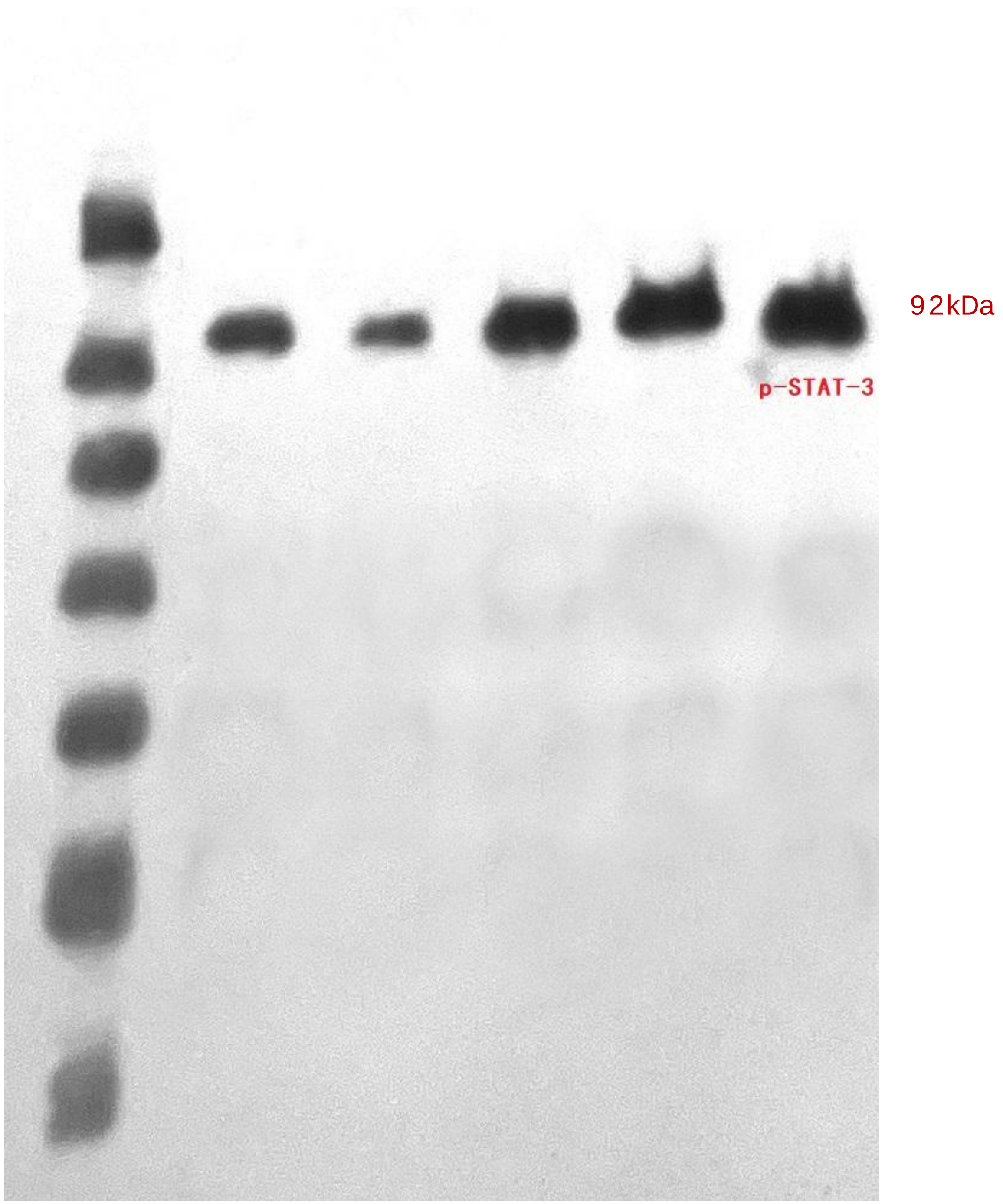
92kDa
p-STAT-3

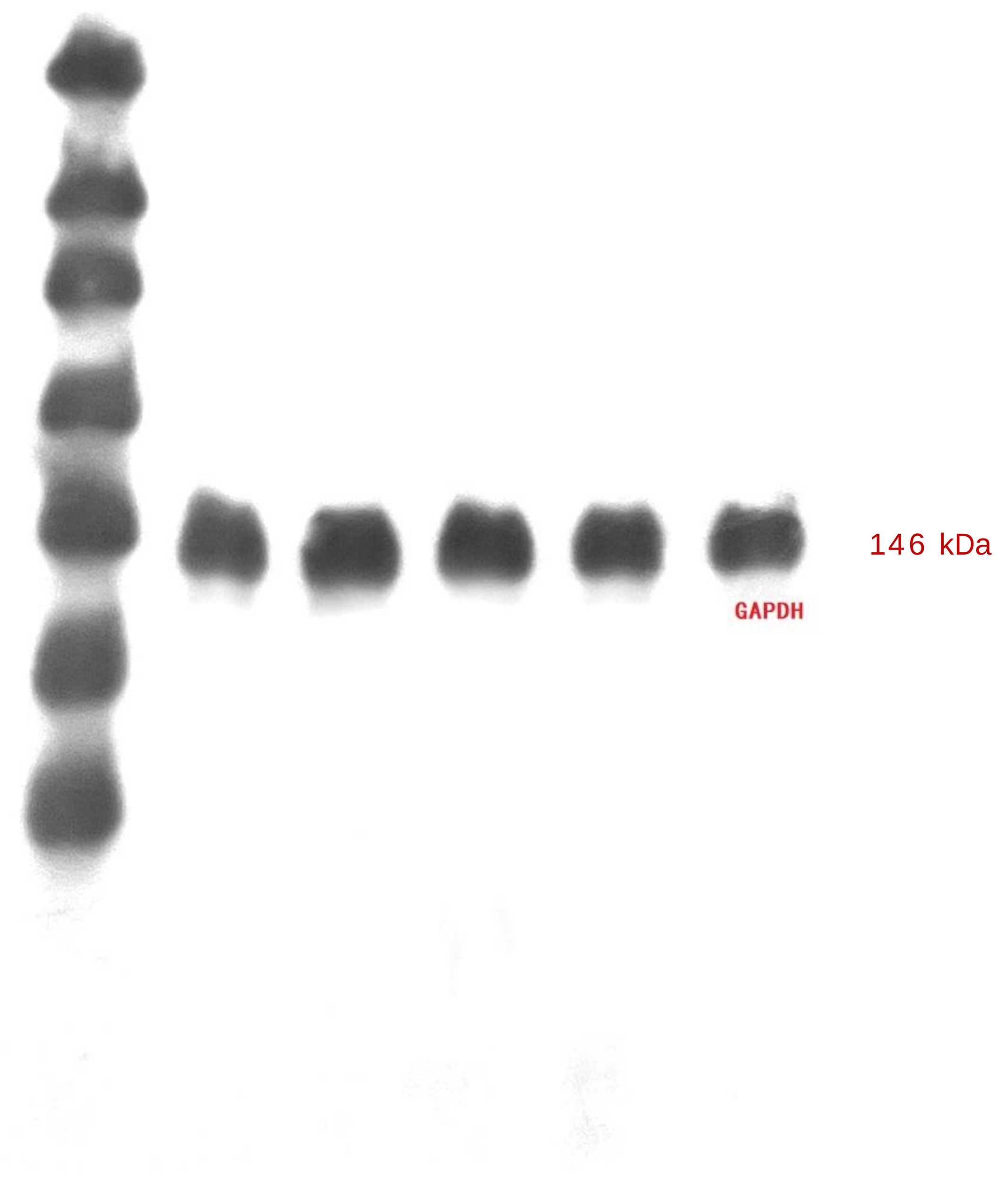
146 kDa
GAPDH

Fig.6A

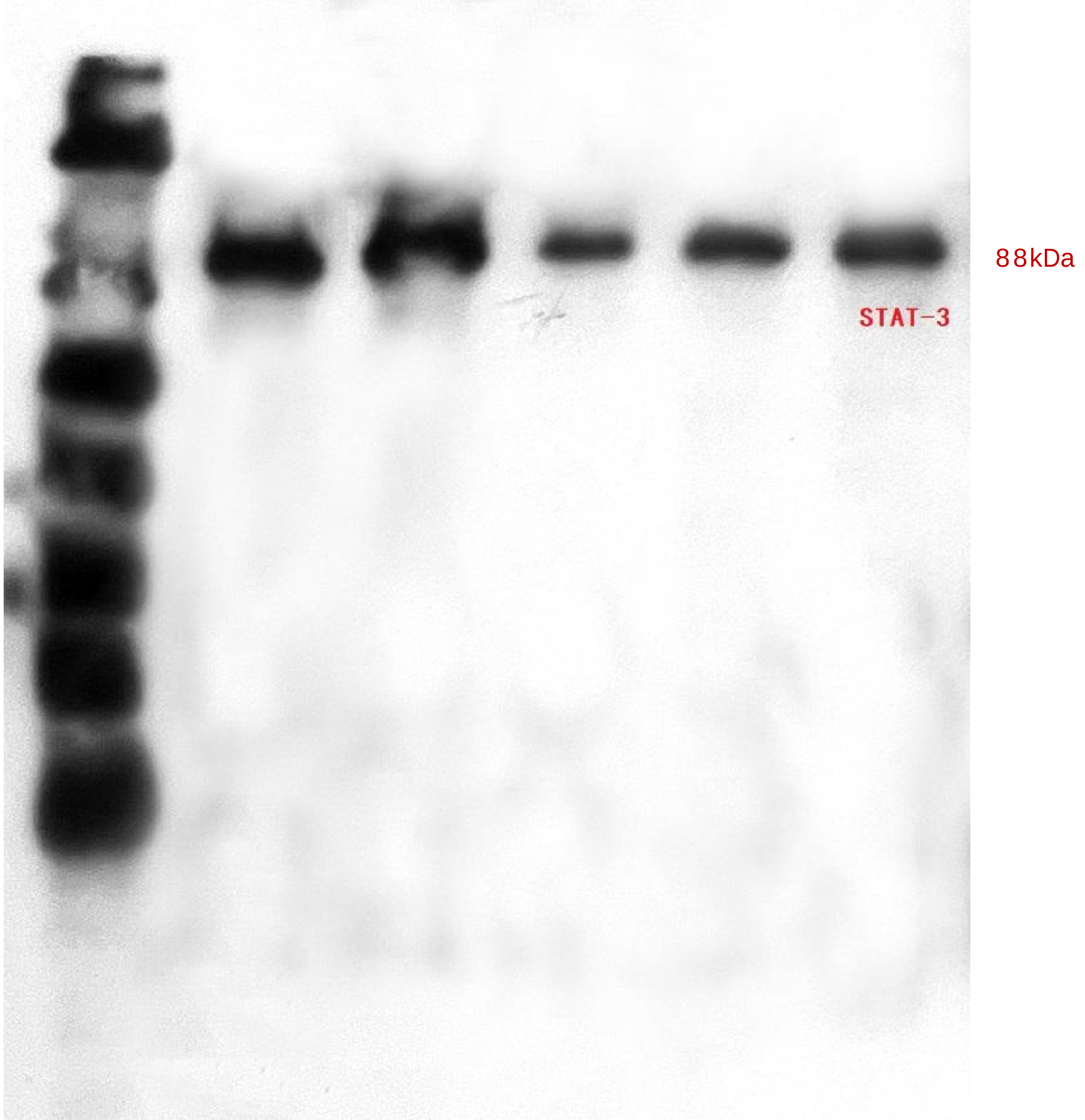

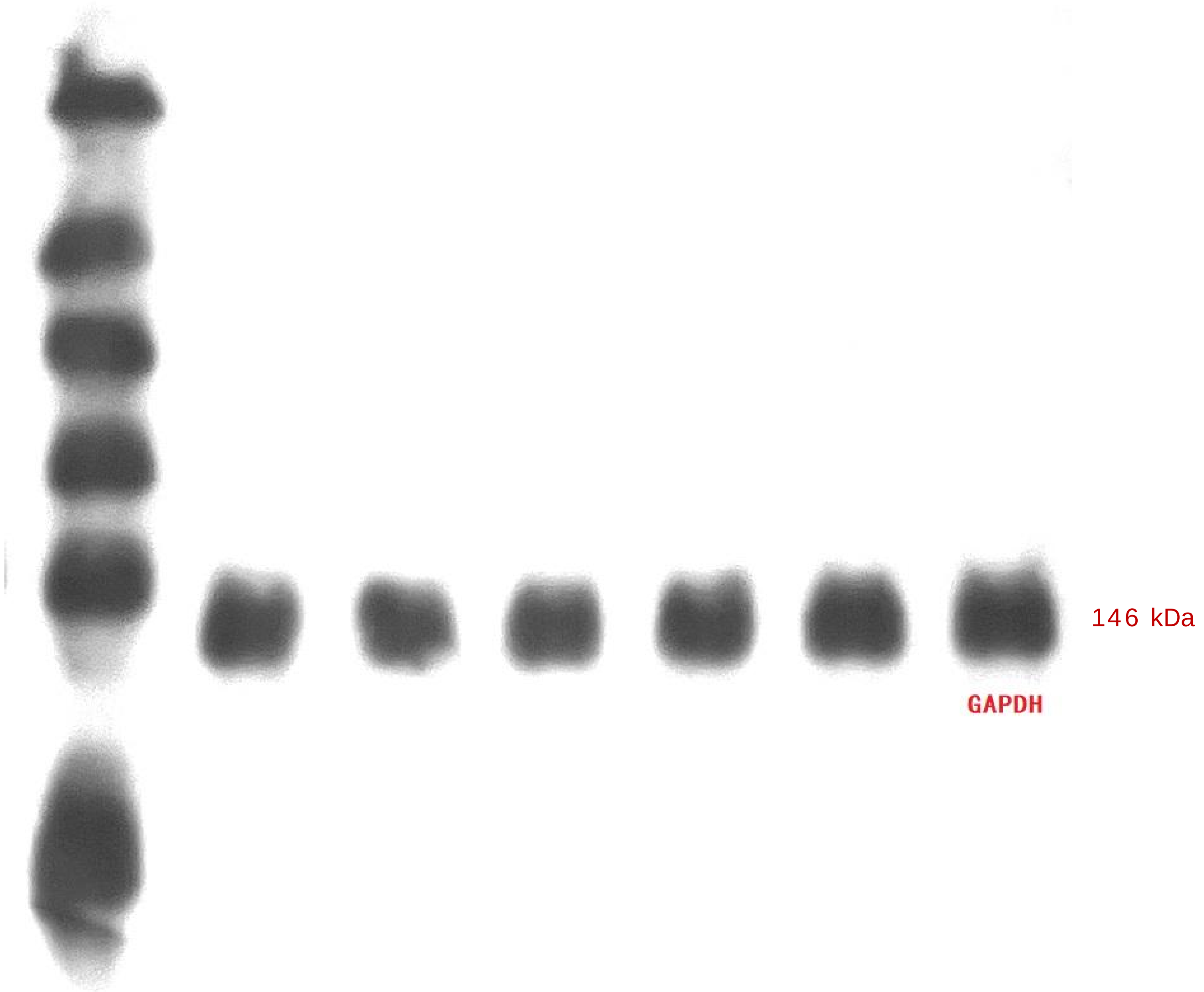
146 kDa
GAPDH

Fig.6C

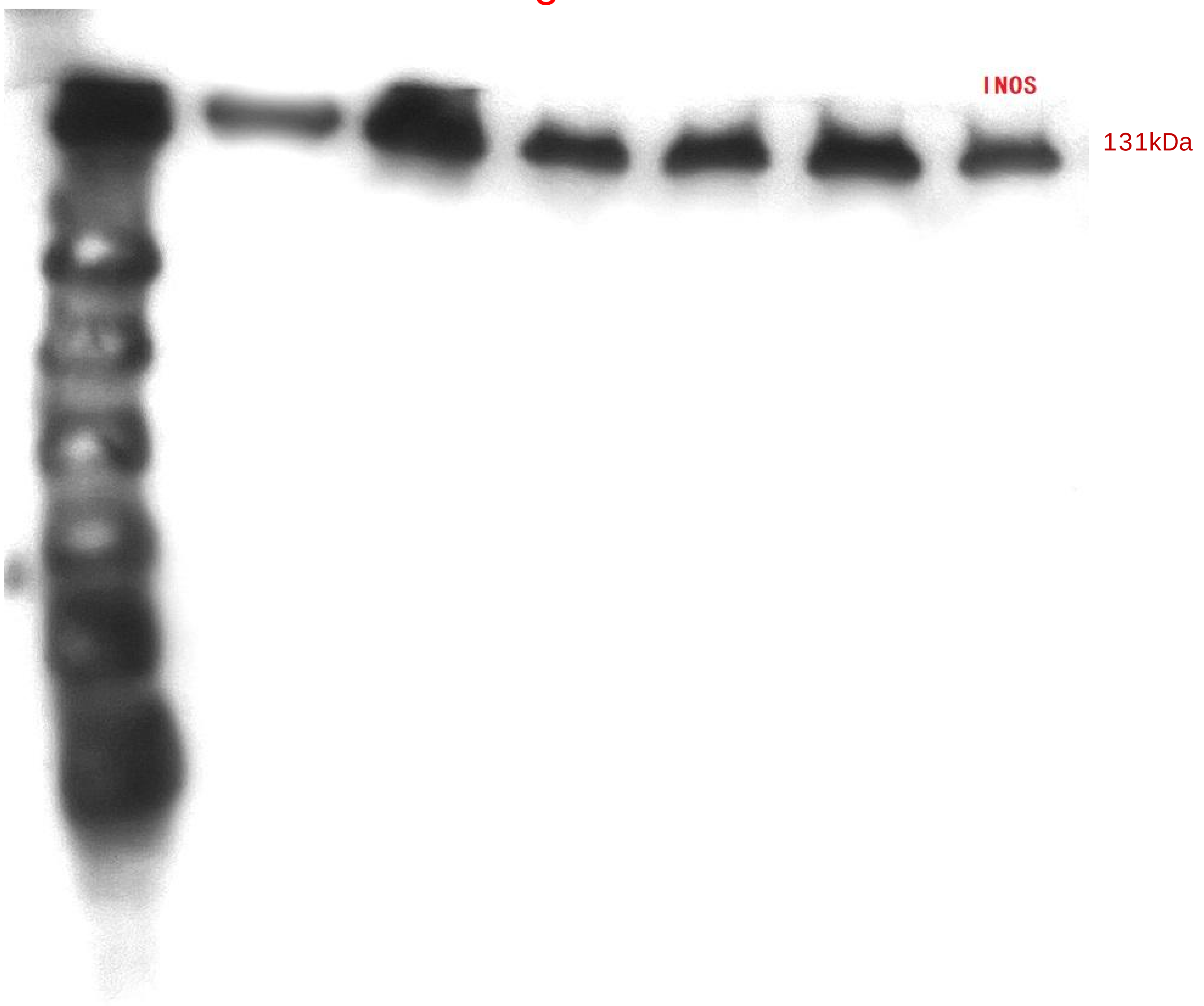

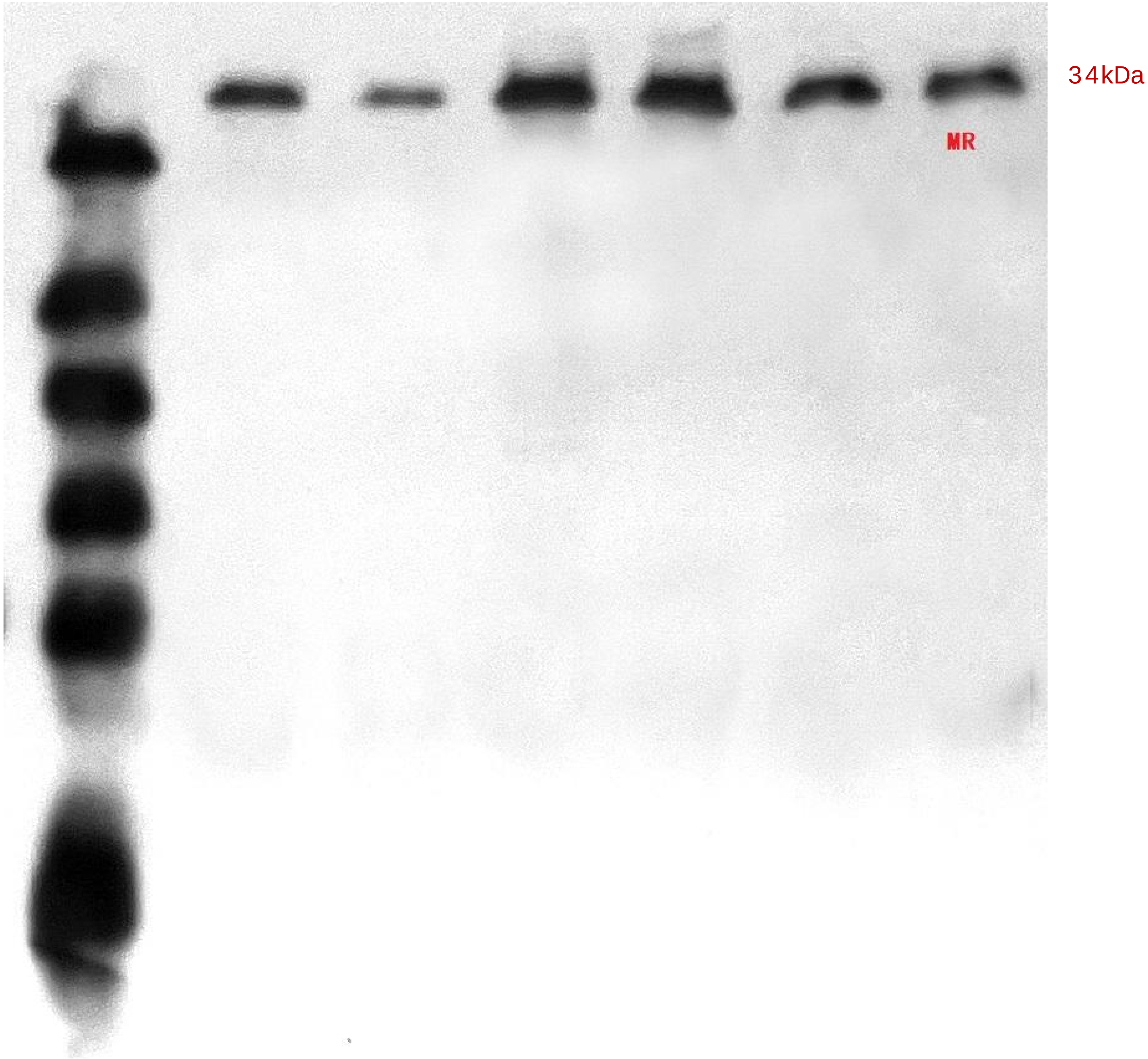
34kDa
MR

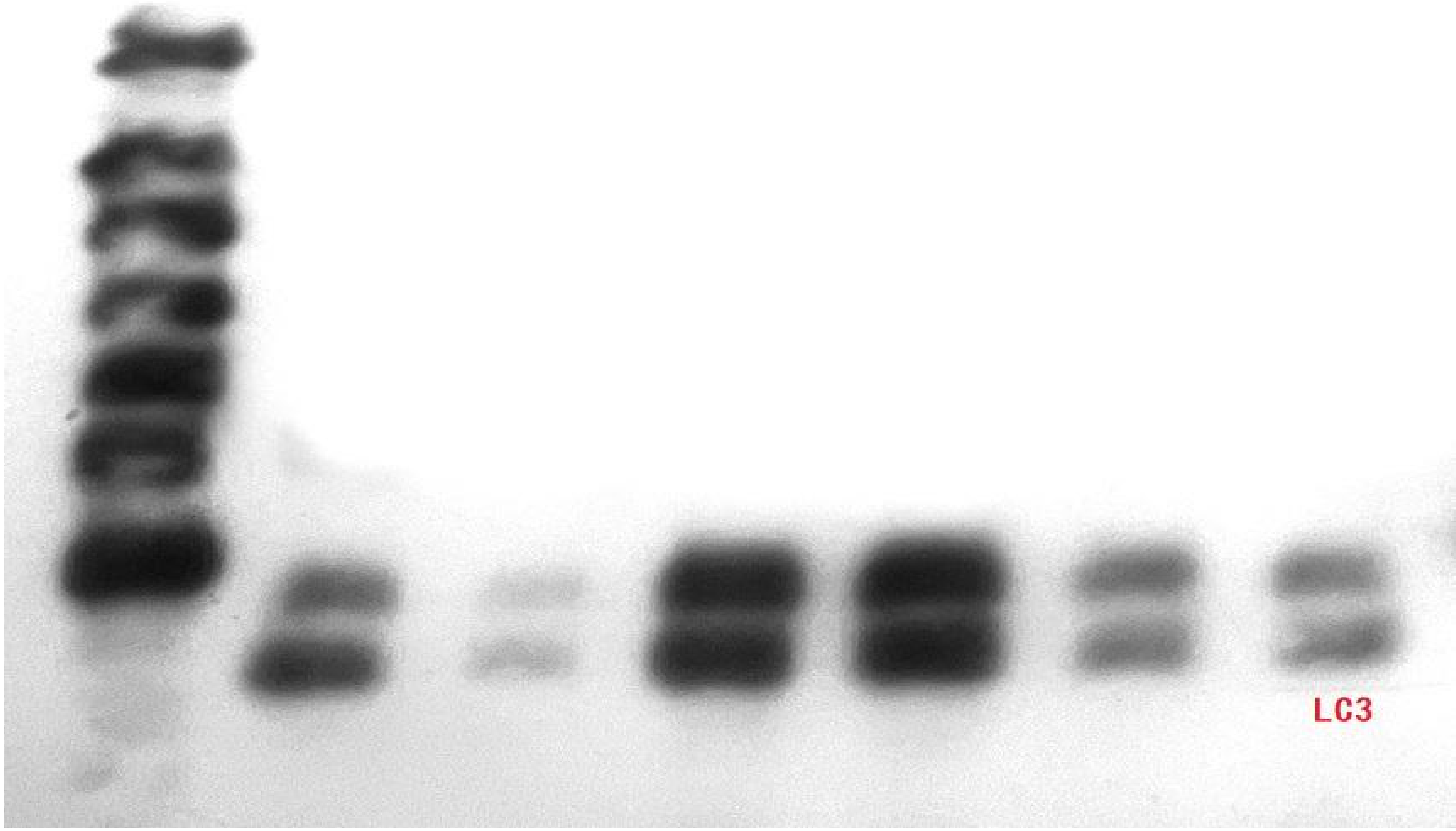

16 kDa

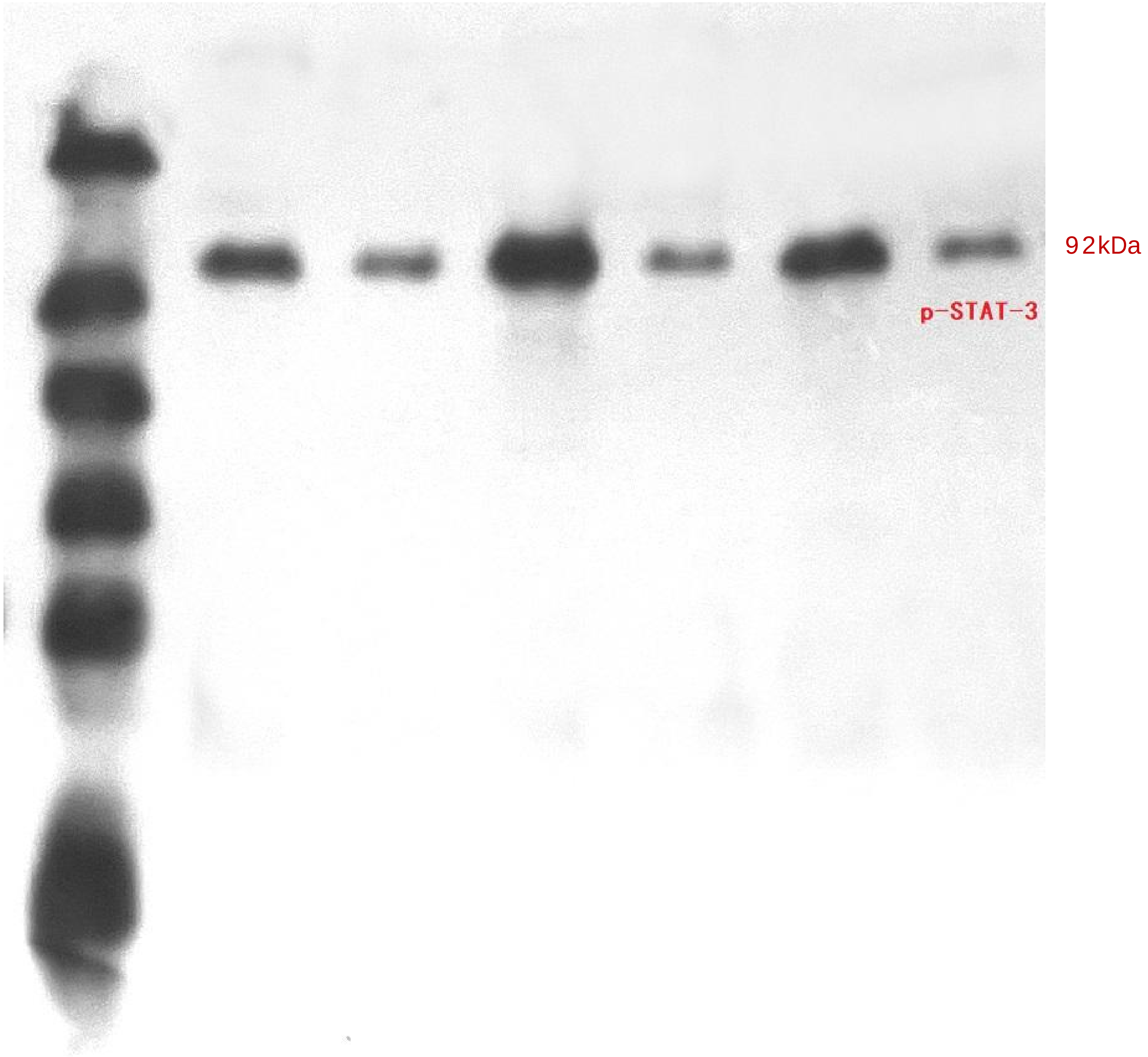
92kDa
p-STAT-3

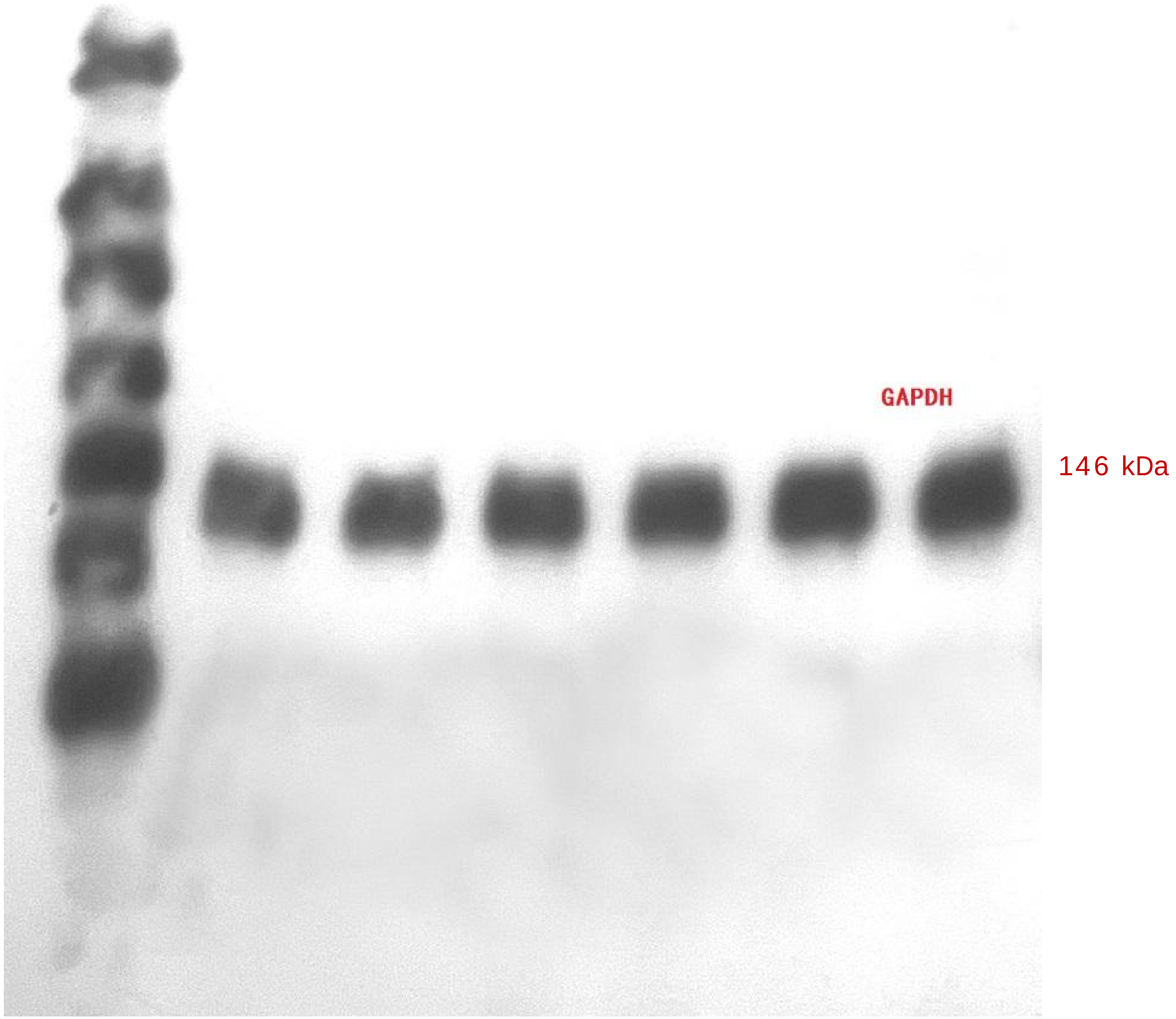
GAPDH
146 kDa
